# Exploring Neural Signal Complexity as a Potential Link between Creative Thinking, Intelligence, and Cognitive Control

**DOI:** 10.3390/jintelligence9040059

**Published:** 2021-11-30

**Authors:** Yadwinder Kaur, Selina Weiss, Changsong Zhou, Rico Fischer, Andrea Hildebrandt

**Affiliations:** 1 Department of Psychology, Carl von Ossietzky Universität Oldenburg, 26129 Oldenburg, Germany; 2 Institute of Psychology and Education, Department of Individual Differences and Psychological Assessment, Ulm University, 89081 Ulm, Germany; selina.weiss@uni-ulm.de; 3 Department of Physics, Centre for Nonlinear Studies, Institute of Computational and Theoretical Studies, Hong Kong Baptist University, Kowloon Tong 999077, Hong Kong; cszhou@hkbu.edu.hk; 4 Department of Psychology, University of Greifswald, 17489 Greifswald, Germany; rico.fischer@uni-greifswald.de

**Keywords:** creative thinking, divergent thinking (DT), creative/fluent verbal association, verbal creativity, fluid (gf) and crystallized intelligence (gc), brain signal complexity (BSC), multi-scale entropy (MSE), cognitive control, inhibition

## Abstract

Functional connectivity studies have demonstrated that creative thinking builds upon an interplay of multiple neural networks involving the cognitive control system. Theoretically, cognitive control has generally been discussed as the common basis underlying the positive relationship between creative thinking and intelligence. However, the literature still lacks a detailed investigation of the association patterns between cognitive control, the factors of creative thinking as measured by divergent thinking (DT) tasks, i.e., fluency and originality, and intelligence, both fluid and crystallized. In the present study, we explored these relationships at the behavioral and the neural level, based on *N* = 77 young adults. We focused on brain-signal complexity (BSC), parameterized by multi-scale entropy (MSE), as measured during a verbal DT and a cognitive control task. We demonstrated that MSE is a sensitive neural indicator of originality as well as inhibition. Then, we explore the relationships between MSE and factor scores indicating DT and intelligence. In a series of across-scalp analyses, we show that the overall MSE measured during a DT task, as well as MSE measured in cognitive control states, are associated with fluency and originality at specific scalp locations, but not with fluid and crystallized intelligence. The present explorative study broadens our understanding of the relationship between creative thinking, intelligence, and cognitive control from the perspective of BSC and has the potential to inspire future BSC-related theories of creative thinking.

## 1. Introduction

Creative thinking and intelligence are vital cognitive abilities for human endeavor, evolution, and cultural transformation (Gabora and Kaufman 2010). Current empirical evidence supports that these two mental faculties are positively associated. However, the mechanism underlying this association is still being explored through the lens of psychometric and neuroscientific investigations. A proposed explanatory mechanism of the relationship between creative thinking and intelligence poses a shared and differential involvement of inhibitory control in both abilities (Benedek et al. 2014). An increasing body of research converges on the notion that inhibition is required for creative thinking as individuals need to inhibit their typical and dominant responses to produce unique, creative ideas. Thus, inhibitory control is necessary to restrain someone’s usual responses, fostering creative idea generation (see Cassotti et al. (2016) for a review). Network neuroscience and latent variable analysis of multiple behavioral performance measures have investigated the mechanisms underlying the relationship between creative thinking and intelligence (Beaty et al. 2016; Kenett and Faust 2019), but hitherto applied functional connectivity measures on magnetic resonance imaging (MRI) data ignore the temporal patterns in neural activities.

The temporal complexity measures of brain signals yield explicative information about the functional activity of neural networks during resting and task-processing states. Studies have demonstrated brain signal complexity (BSC) to be a sensitive neural marker of creative thinking (Kaur et al. 2020; Ueno et al. 2015), healthy brain functioning, knowledge representation (Heisz et al. 2012), cognitive control (Grundy et al. 2019; Wang et al. 2020), and cognitive performance in general (for a review, see Garrett et al. 2013). Nevertheless, it remains to be explored how BSC measures that were captured during different tasks are associated with each other, especially with respect to the creative thinking and inhibition-related neural states. Furthermore, the question remains on how BSC in these states is related to DT, i.e., fluency and originality, and intelligence, i.e., fluid (gf) and crystallized intelligence (gc). Therefore, in the present investigation, we explore the relationship between fluency, originality, gf, gc, and cognitive control (inhibition) at the behavioral and neural levels.

### 1.1. On the Association between Creative Thinking and Intelligence—Behavioral and Neural Evidence

A series of behavioral and neurocognitive studies demonstrated a positive relationship between creative thinking and intelligence (e.g., Benedek et al. 2014; Silvia 2008, 2015). With respect to the behavioral level, considerable effort has been made to link creative thinking with general intelligence (g), for example, as a lower-order factor of g, underneath general retrieval ability (McGrew 2009), but also specifically in terms of gf and gc (Weiss et al. 2020). Robust positive associations between gc and creative thinking (Howrigan and MacDonald 2008; Krumm et al. 2018), and gf and creative thinking (e.g., Benedek et al. 2014) were demonstrated. A recent, fairly large and multivariate study by Weiss et al. (2020) revealed moderate positive latent level relationships between DT, g, and gc. The findings replicated earlier studies on the association between DT and gc (Beaty and Silvia 2012; Silvia and Beaty 2012), suggesting that verbal creativity benefits from the richness of individuals’ verbal knowledge. Many studies also demonstrated an association between DT and gf (e.g., Beaty and Silvia 2012; Benedek et al. 2012, 2014; Silvia and Beaty 2012). Jauk et al. (2013) provided further support for the notion that intelligence is required to put creative idea generation into action (Sternberg and O’Hara 1999).

Network neuroscience has revealed a considerable overlap between brain networks, associated with individual differences in creative thinking and g. The functional network connectivity underlying creative thinking is characterized by interactions between two crucial neural networks: the default-mode network (DMN) and the executive control network (ECN). The former consists of a set of midline and inferior parietal regions considered to facilitate the self-referential thought process, introspection, and imagination, whereas the latter consists of lateral prefrontal and anterior inferior parietal regions underlying externally focused attention and inhibitory control (Beaty et al. 2015, 2016). The dynamics of these two networks were demonstrated to be associated with intelligence. For example, a resting-state functional connectivity study by Hearne et al. (2016) showed greater connectivity between the DMN and the frontoparietal network (FPN) that correlated with higher intelligence scores. The FPN involves hubs instantiating cognitive control, which serves to initiate flexible interactions with other brain networks (Marek and Dosenbach 2018). A study by Cole et al. (2015) found that the across-network connectivity of the lateral prefrontal cortex (a hub of the FPN) predicted gf ability.

Although the DMN and ECN operate in opposition, their coordination during heightened creative thinking and reasoning represents a consistent finding (Beaty et al. 2015, 2016, 2019; Hearne et al. 2015, 2016). As further support, a meta-analysis conducted by Santarnecchi et al. (2017) highlighted the interaction between the attention, salience, and cognitive control networks that serve gf. Additionally, a recent study by Frith et al. (2021a) found that connectome-based predictive models built on fMRI data during a DT task reliably predicted facets of both DT and intelligence. Furthermore, the results revealed considerable overlap in the functional connectivity patterns predicting both abilities. These were predominant within the ECN, salience, and visual networks. Associations were also demonstrated for structural connectivity networks. A diffusion tensor imaging study by Kenett et al. (2018) examined the extent and ways in which cognitive control contributes to creative thinking and intelligence. The authors showed that DT was associated with modal controllability within regions of the DMN-ECN network. Modal controllability characterizes brain regions that require substantial input energy. However, better gf was related with average controllability, which characterizes brain regions that require less input energy. Thus, the authors refer to creative thinking as being a “difficult-to-reach” neural state and gf as an “easy-to-reach” neural state. Overall, DT and gf were associated with the network controllability of regions within the DMN-ECN network, demonstrating that multiple control processes were involved in creative thinking and intelligence. These results are consistent with previous studies that demonstrated a substantial role for DMN–ECN interaction in creative thinking, as well as in gf (Beaty et al. 2015, 2016; Hearne et al. 2015, 2016). Furthermore, the results converge with the abundant literature pointing to the involvement of the prefrontal cortex (PFC) in creative thinking (see Dietrich and Kanso (2010) for a review), suggesting that the dynamic modulation of cognitive control, driven by PFC networks, is potentially its key mechanism (Chrysikou 2018; Dietrich 2004).

### 1.2. On the Role of Cognitive Control in Creative Thinking and the Creative Thinking–Intelligence Relationship

Cognitive control is the process by which automatic responses are inhibited and flexibly adapted to produce complex goal-directed thoughts (Morton et al. 2011). It encompasses multiple facets, one of which is inhibition, the process that guards the cognitive system against salient but irrelevant stimuli (Jones et al. 2016). Thus, the ability to inhibit an internal tendency or to restrain from external information is generally referred to as inhibition ability (Xie et al. 2017). The strongest argument linking cognitive control and creative thinking is related to inhibitory control. The following working mechanism has been proposed: the inhibition of common ideas and the later evaluation and monitoring of produced responses lead to improved unusual idea generation (Beaty et al. 2014; Nusbaum et al. 2014; Nusbaum and Silvia 2011). Thus, individuals need to inhibit their irrelevant or common responses to create novel ideas (Benedek et al. 2012; Camarda et al. 2018; Zabelina and Ganis 2018).

Another line of theoretical argument proposes that the cognitive basis of creative thinking relies rather on the joint contributions of associative and executive abilities. For example, a study by Beaty et al. (2014) explored how associative and executive processes relate to creative thinking. To this end, the study measured the semantic distances of responses generated during verbal fluency tasks, broad retrieval ability, and gf. By means of structural equation modeling (SEM), the authors showed that the associative abilities indicated by semantic distance and individual differences in broad retrieval ability and gf can predict DT ability. Moreover, cognitive control has been shown to partly explain the relationship between creative thinking and intelligence. By using a latent variable-modeling approach, Benedek et al. (2014) examined whether inhibition, updating, and shifting explain the relationship between the originality facet of creativity and gf. They showed that gf was significantly associated with updating, but not with shifting and inhibition. Creative thinking was predicted by updating and inhibition, but not by shifting. Furthermore, the study revealed that accounting for these executive functions in gf and creative thinking diminished the association between the two. In a similar line of research, an even earlier study by Nusbaum and Silvia (2011) considered switching between categories during a DT task as an executive control ability (Gilhooly et al. 2007). The assumption is that switching requires us to overcome interference within category associations. The study showed that the effect of gf on DT was mediated by the switching ability, supporting the premise that executive abilities play a substantial role in DT performance. Similarly, Frith et al. (2021b) recently found considerable overlap between DT and gf at the level of latent abilities, which was, however, explained by executive attention.

To conclude, there is considerable empirical evidence showing that certain executive functions (inhibition, updating, and executive attention) are indeed critical to creative thinking (Diamond 2013; Frith et al. 2021b) and that they also seem to play an explanatory role in the creative thinking–intelligence relationship. The aforementioned role of cognitive control in DT, demonstrated at the behavioral level, delivers an explanation for the involvement of extensive brain networks, such as the DMN and ECN, and local PFC in both cognitive control and DT. These widely distributed and local networks that serve creative thinking seem to encompass two contradictory neurocognitive states. One interpretation of their simultaneous engagement during creative thinking has been offered in a recent review by Chrysikou (2018). The author emphasized the importance of the bottom-up and top-down processes involved in creative idea generation. The former processes rely on the activity of the DMN and contribute to making unanticipated conceptual associations relevant to the idea generation phase. The latter processes rely on the PFC and retain the evaluations of significance, feasibility, viability, and efficacy of the produced associations along the bottom-up loop. Thus, there seems to be an iterative switching between bottom-up or spontaneous and top-down or controlled processing steps that is argued to be essential for creative thinking.

Another line of research reported brain activations during DT tasks to be associated with cognitive control. For example, Zabelina (2018) reviewed studies showing activations in the inferior frontal gyrus during DT task performance to be associated with cognitive control (Abraham 2014; Benedek et al. 2014). A recent review by Zhang et al. (2020) describes a mechanistic approach to understanding creative thinking by comparing and discussing the neural correlates of convergent thinking and DT. The review provides a theoretical integration, suggesting that creative cognition, as measured by DT and convergent thinking tasks, is modulated by meta-control states. Specifically, they suggest that the activation dynamics of the left inferior frontal gyrus facilitate DT and insight solutions. In summary, behavioral and neural evidence seem to support that executive functions, including cognitive control, are integral to creative thinking because they support novelty-seeking, evaluation, and assessment of more or less creative ideas.

### 1.3. Brain Signal Complexity (BSC) as a Neural Marker of Creative Thinking and Intelligence

Brain signal complexity (BSC) arises from the interaction among numerous neuronal circuits that operate over a wide range of temporal and spatial scales. These spatiotemporal fluctuations reveal important underlying dynamic information about the brain. The complexity of the EEG signals contains information about the architecture of the neural networks at multiple spatiotemporal scales. This is to say that the inherent properties of a complex system are manifested in the features of the signals produced by it (McDonough and Nashiro 2014; Ueno et al. 2015). Therefore, an understanding of the complex fluctuations of neural signals can capture essential underlying features describing the functioning of the system. Thus, the analysis of the underlying complexity of EEG signals has been argued to provide powerful insights into human brain functioning.

BSC has been previously studied by means of entropy-based methods that quantify the complexity, uncertainty, or irregularity of a complex system’s activity (Sandler 2017) manifested in a time series, such as the brain’s electrical signals. Thus, entropy-based methods are appropriate concepts to estimate neural signal complexity. In the context of information theory (Shannon 1948), entropy refers to the information content of a system. Thus, a physiological signal with higher entropy is taken to indicate a higher information-processing capacity (Heisz and McIntosh 2013). According to the conventional interpretation, low entropy is assumed to characterize a highly predictable system that is less complex, and high entropy is assumed to reflect a less predictable signal originating from the system.

Multi-scale entropy (MSE) (Costa et al. 2002, 2005) provides temporal complexity estimates over a range of multiple spatiotemporal scales. The MSE computation relies on the sample entropy calculation (SampEn) (Richman and Moorman 2000), but it additionally divides the original signal into coarse-grained time series (for more details, see the Materials and Methods section) taken to indicate system dynamics on different temporal scales. These temporal scales are broadly divided into small and large scales when interpreting entropy measures. Entropy at small temporal scales yields information about local neural processing and characterizes the irregularity of the high-frequency dynamics, whereas large temporal scales are considered to represent joint activity across more widely distributed networks and to characterize low-frequency dynamics (Courtiol et al. 2016). Thus, taking different scale levels together, MSE estimates reveal the relative contributions of local and global information processing in the brain (Grundy et al. 2017; McIntosh et al. 2014; Vakorin et al. 2017). In summary, MSE provides a quantitative index of a dynamic system’s irregularity. It assigns large values to more complex signals and small values to highly deterministic signals (Costa et al. 2005). In general, signals such as neural oscillations and stationary signals that feature a repetitive structure are allocated lower entropy and, hence, lower complexity. In contrast, highly irregular or non-repetitive signals (less predictable) are assigned higher entropy and, hence, higher complexity.

Several studies have estimated BSC by means of MSE and demonstrated that it is a neural marker of healthy brain functioning, cognitive performance, typical development, and knowledge representation, among others (Garrett et al. 2011, 2013; Heisz et al. 2012; Lippé et al. 2009; McIntosh et al. 2008; Mišic et al. 2010). Hence, MSE is a well-established neural marker of cognition. The MSE of temporally and spatially distributed brain activity (as measured via EEG or fMRI) acquired during resting or task performance states have also been related to individual differences in creative thinking and intelligence; however, this is in separate studies. For example, a study by Ueno et al. (2015) showed higher MSE in a resting-state EEG across large temporal scales in more creative compared with less creative elderly individuals. A resting-state fMRI study by Shi et al. (2020) showed regional Blood oxygenation level dependent (BOLD) signal entropy to be positively correlated with DT. Furthermore, the authors pointed out that creative thinking was closely related to the functional dynamics of the cognitive control network involved in cognitive flexibility and inhibition. Other studies on brain signal entropy in resting-state fMRI also showed substantial associations with gc (Saxe et al. 2018). Moreover, information from cortical entropy profiles effectively predicted several cognitive abilities in a recent study by Li et al. (2020).

Importantly for the aims of the present study, Kaur et al. (2020) showed that BSC, as indexed by MSE, was higher in those neural states in which individuals were asked to produce unusual, creative (as opposed to usual), fluent verbal associations. This was the first evidence to prove BSC as a neural state marker of creative verbal associations. Based on the above literature and our previous finding, we thus infer that creative thinking, gf, and gc are all associated with neural signal complexity to some extent. The question is, by how much are these associations distinct, and which cognitive states contribute to their distinction? Can BSC measured during cognitive control explain the fact that BSC was discovered to be a neural marker not only of creative thinking but also of reasoning? Can neural complexity provide insights into the associations between gf, gc, fluency, and originality? Given the theorized role of cognitive control in creative thinking and the creative thinking–intelligence relationship, we expect MSE in cognitive control brain states to provide supportive evidence.

### 1.4. Aims of the Study

In line with the evidence outlined above, we assume that the relationship between creative thinking, intelligence, and cognitive control is due to the task-dependent synchronized co-activation of several local and largely distributed brain networks, including the FPN, DMN, ECN, and salience networks. Thus, in line with the literature, we argue that the investigation of the interrelation between these complex mental abilities and their underlying neural mechanisms needs also to rely on methods capturing temporal neural complexities that characterize those brain networks. MSE is thus a candidate measure because it captures neural complexity across local and widespread neural networks. Investigating individual differences in the EEG-derived MSE and behavioral performance outcomes indicating creative thinking, intelligence, and cognitive control, as well as their relationships, will thus provide new and complementary insights into the neural foundations of the creative thinking–intelligence relationship. To date, there is no study that has directly examined the relationship between MSE measured during both creative thinking and cognitive control tasks. Furthermore, given the notion that cognitive control partly or fully explains the relationship between creative thinking and intelligence, it remains to be investigated whether MSE captured during creative thinking and inhibition are differentially associated with the behavioral outcomes of gf, gc, and DT tasks, measuring fluency and originality.

For this study, we acquired the behavioral measures of gf, gc, fluency, originality, and inhibition, as well as brain signals derived from an EEG during the performance of a verbal DT (creative thinking measure) task and a cognitive control (inhibition measure) task. The verbal DT task required participants to produce unusual or creative and fluent or typical verbal associations. We also labeled MSE during creative verbal association production as MSE in creative idea generation, and MSE during typical verbal association production as MSE in fluent idea generation. The cognitive control task consisted of inhibition (i.e., high-control states) and non-inhibition (i.e., low-control states) conditions. Generally, in line with Benedek et al. (2014), we expect that inhibition would partly account for the creative thinking–intelligence relationship at the behavioral level. Furthermore, a previous study (Kaur et al. 2020) has shown that the MSE in different task conditions of verbal creativity (i.e., during productions of original vs. typical associations in a verbal DT task) were different on average but were highly correlated. However, different task types (e.g., creative thinking vs. inhibition) involve different mental operations, and signal complexity during such task states is expected to show weaker relationships. We expect that individual differences in MSE measured during the production of creative verbal associations are more strongly associated with MSE during inhibition, compared with the associations between inhibition MSE and MSE measured during typical association production. This is because, theoretically, the generation of original associations implies inhibiting the usual associations (see above). However, fluency also implies inhibiting incorrect associations; thus, a relationship between MSE in inhibition and MSE for the usual associations can be expected as well. Furthermore, we aim to explore here the relationships between the MSE indicators of creative thinking and cognitive control with gf, gc, fluency, and originality, measured via independent behavioral tasks. Taking all the above into consideration, in this exploratory study we aspire to answer the following research questions:

1. Does the ability to inhibit irrelevant information explain the positive association between fluency/originality and intelligence (gf and gc)? The previous research reviewed above suggested that the relationship between originality and gf can be partially or fully explained by inhibition ability. Here we aim to extend these findings by considering multiple facets of intelligence and creative thinking, thus also including fluency and gc.
2. Is MSE higher in low-control states (non-inhibition) compared with high-control states (inhibition)? In line with previous findings that, especially at higher scales, MSE depletes with the increasingly focused demands of the neural system, we expect to affirm this question. Should this be the case, we would conclude that MSE can be considered a neural marker of inhibition. This is a requirement for investigating MSE in inhibitory brain states as a correlate of fluency and originality.
3. Is there a positive association between MSE captured during a verbal DT task and MSE measured during inhibition states? Producing original verbal associations theoretically implies inhibiting the typical associations that occur more or less automatically. We thus expect MSE in creative verbal association production states to be more strongly associated with MSE in inhibition states, compared with MSE measured during the production of typical verbal associations.
4. Is MSE in inhibition states, and MSE measured during the production of original associations, more strongly associated with fluency and originality, compared with gf and gc abilities?

## 2. Materials and Methods

### 2.1. Sample and Procedure

The measurements applied in the present study were approved by the ethics committee of the Department of Psychology, Humboldt-Universität zu Berlin. Participants signed informed consent before participating in the experiments. Data were assessed in two independent sessions: (1) behavioral tasks session, (2) EEG recording session. In the first session, we acquired behavioral estimates of gf, gc, fluency, and originality from *n* = 159 participants. In the second session, the same participants performed two different tasks while the EEG was recorded. There was a verbal DT task (completed by *n* = 101) and a numerical Simon task (completed by *n* = 90), both of which were involved in the MSE analyses. All tasks applied in both sessions were programmed with PsychoPy (Peirce and MacAskill 2018). To test brain–behavior relationships, the datasets collected during the behavioral and the EEG sessions were merged. Participants with fewer than 10 years of German language-speaking experience were excluded. After the exclusion, and given the samples available for the different measurements, the final sample included *N* = 77 young adults (34 females, *M* age = 23.80 years, *SD* age = 3.79, range = 18–32). Among them, 5 had not obtained high-school qualifications, 58 had high-school or equivalent qualifications, and 14 had academic degrees (e.g., Bachelor’s, Master’s or Diploma). The dataset and related results of the current study are available online via OSF (https://osf.io/sg9e2/ Accessed on: 15 November 2021).

### 2.2. Tasks Performed in the Behavioral Session

#### 2.2.1. Factors of Creative Thinking Measured by DT Task: Fluency and Originality

We used four measures of verbal fluency and two measures of verbal originality. The two measures of verbal fluency were adapted from the verbal creativity test (Verbaler Kreativitäts-Test-VKT; Schoppe 1975), namely, similar attributes (SA), (e.g., “Name as many things as possible that are inedible for humans”) and inventing names (IN) (e.g., “Invent as many names as possible for the abbreviation: ‘T-E-F’“). SA and IN tasks required participants to produce as many context-appropriate answers as possible within 60 s. The SA task consisted of six items and the IN task consisted of 18 items. The third measure was retrieval fluency (RF) (e.g., “Name as many things as possible that are suitable for the given category”) which was adapted from the kit of factor-referenced cognitive tests (Ekstrom and Harman 1976) and was translated from English to the German language. Furthermore, we adapted four figural fluency (FF) tasks from the *Berliner Intelligenzstruktur-Test für Jugendliche: Begabungs- und Hochbegabungsdiagnostik* (Berlin Structure-of-Intelligence Test for Youth: Diagnosis of Talents and Giftedness; Jäger et al. 1997). This series of tasks required that participants draw objects (using paper and a pencil) based on different shapes (e.g., “Draw as many shapes as possible from a given rectangle and circle”) and to come up with a creative figural emblem or logo for a company. The four tasks lasted for 1:50, 3, 1:45, and 3 min respectively.

Verbal originality was assessed using two tasks, namely, combining objects (CO) (e.g., “Combine two objects to build a door stopper in your house”) and nicknames (NI) (e.g., “Invent a nickname for a shirt”). For each of the tasks, participants were instructed to provide a single answer that was unique and original. The CO task was adapted from the kit of factor-referenced cognitive tests (Ekstrom and Harman 1976) and consisted of 12 items (with a time limit of 60 s per item, translated from English to the German language). The NI task was adapted from the VKT (Schoppe 1975) and included 9 items with a time limit of 30 s for each item.

#### 2.2.2. Measured Facets of Intelligence: Fluid (gf) and Crystallized Intelligence (gc)

Intelligence was assessed within the figural and verbal content domains. We adapted three reasoning tasks from the Berlin Test of Fluid and Crystallized Intelligence (BEFKI) (Wilhelm et al. 2014). The verbal part of fluid intelligence (gfv) consisted of 16 items that required participants to answer questions based on relational reasoning (e.g., “If Frank is bigger than Hans, who is the smaller of the two?”). The figural part (gff) required participants to assess how a sequence of geometric drawings, created based on different rules, should continue. Both of these tasks had a time limit of 14 min each. The crystallized intelligence (gc) tasks assessed knowledge from three broad domains: natural sciences (gcnature), humanities (gchuman), and social studies (gcsocial), using a 32-item test that lasted for 10 min. Furthermore, gc items were parceled out according to their domain, to be used for statistical analyses.

### 2.3. Tasks Performed in the EEG Recording Session

The recording of the EEG datasets during the performance of a verbal DT and a numerical Simon task took place in a closed, quiet, and well-illuminated room, using the Brain Vision Recorder software (Brain Products GmbH, Gilching, Germany). We used BrainAmp DC amplifiers (Brain Products, Germany) to amplify the EEG signals, with an amplitude resolution of 0.1 μV at a sampling rate of 250 Hz. Cutoff filters were 0.16 and 1000 Hz at the low and high range, respectively. An EEG cap (Easycap, Brain Products, Germany) was fitted with 30 Ag/AgCl electrodes, placed according to the 10–20 system. Eye movements and blinks were monitored using electrodes positioned at the outer canthi of both eyes and below the right eye. The A1 electrode (left mastoid) was used as a reference, and AFz served as ground. Impedances were kept below 5 kΩ.

#### 2.3.1. The Verb-Generation Task

To assess creative thinking, we used the original verb generation task (adapted from Prabhakaran et al. 2014) which was modified by translating the stimulus material into the German language (for the task paradigm and further details, see Kaur et al. 2020). The task required the participants to produce a verb that is semantically related to the presented noun stimulus. The nouns were cued to two colors: purple and green. In total, there were 35 purple-cued nouns and 32 green-cued nouns. For purple-cued nouns, participants were expected to produce usual or typical associations—we thus instructed them to type in the verb that first came to their minds when being presented with the noun (fluency condition). For green-cued nouns, participants should produce original, unique verb associations in response to the noun (originality condition). The role of explicit instructions, e.g., “be creative” or “be fluent”, has been recommended in the creativity research and is hypothesized to increase the relationship between intelligence and DT (Gerwig et al. 2021; Silvia 2015). In the following analysis, we label the two conditions as “typical associations” and “original associations”. The task began with verbal instructions, followed by an example trial and five practice trials. Participants were instructed to type in only one associated verb for each presented noun. The onset of the stimulus and the onset of the participant’s typing response were time-marked, to be taken as signals of interest in the context of MSE analysis. We did not impose any time restrictions during the task, in order to capture the entire cognitive process and the associated brain activity. The EEG recording for the task lasted around 20 min, depending on the participants’ time investment. 

#### 2.3.2. The Numerical Simon Task

To measure inhibition, we used a number-version of the Simon task (Fischer et al. 2008; Plessow et al. 2011). Participants were required to categorize numbers (1–9, except 5) as smaller or larger than five (i.e., identity as a task-relevant stimulus dimension). Participants responded with the left key (Alt on a standard QWERTY keyboard) and the right key (Alt Gr) to numbers smaller and larger than five, respectively. Besides this, all numbers were randomly presented either to the left or to the right of a fixation cross. The task-irrelevant stimulus location automatically activates the spatially corresponding response hand. In compatible conditions, this automatic response activation corresponds to the required response (e.g., number 9 being presented on the right side). Incompatible conditions reflect a mismatch between automatic location-triggered response activation and the required response (e.g., number 1 being presented on the right side). In this situation, top-down control is needed to suppress the incorrect automatic response activation (e.g., a spatially corresponding right response) in order to correctly execute the required response (e.g., a left response to the number 1).

The task began with a practice block (16 trials). Each trial started with a fixation cross presented for 1000 ms, after which the target was added for 200 ms. The fixation cross lasted until a response was given or for a maximum of 1600 ms. For every correct response, a blank screen was shown; for a missed response and for an incorrect response, “falsch (false)” was shown as feedback. Afterward, the blank screen was presented for a random interval of between 100 and 1000 ms. Participants performed three blocks^1^ of trials, including 80% compatible and 20% incompatible trials (96 and 24 trials per block). A high frequency of compatible trials increases the reliance on automatic response activation because the irrelevant stimulus location corresponds with the required response for most of the time. In rare, incompatible trials, however, strong reactive inhibition is needed to control the incorrect automatic response activation triggered by the stimulus location. The difference between incompatible minus compatible trials denotes the Simon effect and represents a marker of stimulus–response conflict. Reaction times (RTs) and accuracies were recorded for each trial. The inhibition scores for modeling the behavioral performance on this task were obtained as RT difference scores between incompatible and compatible conditions (i.e., mean RT in incompatible trials minus mean RT in compatible trials). These were rescaled so that the difference of the RT between the inhibition and non-inhibition trials presents a reverse measure of inhibition (i.e., disinhibition). Thus, we expect that individuals with a larger difference can inhibit less, and individuals with a smaller difference would inhibit more. The difference scores of the RTs were used as indicators in the path analysis. The EEG recording for the Simon task lasted approximately 12–15 min.

### 2.4. Data Processing

#### 2.4.1. Human Ratings of Responses in the DT Tasks

1. The behavioral DT data analyzed here were collected in a multivariate study and partially analyzed by Weiss et al. (2020). Thus, for further information on scoring and details on the DT tasks, we refer to the previous study. The tasks were open-ended; hence, the responses required human coding. Therefore, three human coders were recruited who were semi-experts (psychology students) regarding the analysis of creativity and who went through a training procedure prior to working on the ratings (following CAT; Amabile 1982). The procedures were explained as follows. Fluency (SA, IN, FF, RF)—For the SA and IN tasks, the raters applied a typical fluency rating, i.e., they counted the number of correct answers. The intra-class correlations (ICCs; Shrout and Fleiss 1979), measuring consistency across the fixed set of raters for all items of SA, ranged between 0.96 and 1.00; for IN, this was 0.93–0.98. For the FF and RF tasks, the raters followed the test manual instructions on coding; the ICCs ranged between 0.89 and 0.99, and between 0.99 and 1.00, respectively. For each task, the ratings of the different raters were aggregated, resulting in a single mean score per item. Next, scores across items were also aggregated, to derive a single task score each for SA, IN, FF, and RF, which served as indicators for latent variable modeling.
2. Originality (CO, NI): Every single response from the originality tasks was independently rated by each rater on a five-point scale, based on proposed scoring guidelines from the literature (Silvia 2008; Silvia et al. 2009). A response was rated as original if it was novel (uncommon), remote, or unexpected (clever), compared to the rest of the sample (Silvia 2008). The raters were instructed to rate verbal creativity in relation to the answers given by other participants. Missed or inappropriate answers were rated as zero. Missing values in single tasks were taken as being missing completely at random (n_max_ = 5 (6.5%), n_mean_ = 2.67 (3.5%)). The ICCs for originality were lower compared to the fluency scores but were acceptable. The ICCs for the task, for CO, and for NI were between 0.56 and 0.90. After estimating the ICCs, a compound score was calculated across all three raters for every item, which served as indicators for the originality latent factor.
3. Verbal DT Task: For the verbal DT task applied during the EEG recording, three trained native German speakers also rated all responses; for detailed information on the ratings, please see Kaur et al. (2020). The results of the ratings showed that the individuals indeed produced more creative verbs in the original, compared to the typical, association condition.

#### 2.4.2. Pre-Processing of the EEG Datasets

The EEG data were preprocessed in MATLAB and were filtered offline using IIR (zero phase shift), and Butterworth filters of between 0.1 and 50 Hz (order = 2; time constant = 1.59 s) and recalculated to average reference using the Brain Vision Analyzer (Brain Products, Germany). Further pre-processing steps were executed in EEGLAB (Delorme and Makeig 2004). The artifacts due to blinks and eye movements were handled by applying independent component analysis (ICA) (function: *runica*). Furthermore, SASICA (an EEGLAB plugin (Chaumon et al. 2015)) was used as a guide to select artifactual components.

#### 2.4.3. Multi-Scale Entropy Analyses

MSE is an information-theoretical metric that characterizes the variability of temporal signals, i.e., EEGs across multiple temporal scales, from a perspective on signal complexity (Costa et al. 2002, 2005). The basic rationale of MSE is that multi-scale analysis provides more detailed insight into the underlying biological processes compared with single-scale methods (e.g., approximate entropy). The multi-scale approach approximates the system dynamics on different time scales, which are then analyzed with the SampEn algorithm. There are two critical parameters in the algorithm: *m* defines the length of the signal patterns that are compared with each other, and *r* represents the similarity bounds within which the signal patterns are matched. The MSE algorithm consists of two steps: (1) the coarse-graining of the signal time series at multiple time scales, which means averaging the data inside a window of length τ, in order to reduce the high-frequency components. Coarse-graining is implemented by replacing the progressively increasing number of data points in non-overlapping windows by their average values, to form a new time series (for an illustration, see Costa et al. 2002; Kaur et al. 2019, 2020). The coarse-grained time series at time-scale 1 is identical to the original signal; at scale i, the time series is divided into non-overlapping windows to be concatenated, each of which contains i points corresponding to the time scale. (2) SampEn is then calculated for each of those coarse-grained time series. SampEn identifies the repetition of sequence patterns in the time series and calculates entropy as follows: the number of patterns with i data points, satisfying the similarity bounds *r,* needs to be identified. These are counted and denoted as N(i). Then, the number of similar sequences with i + 1 data points are counted and denoted as N(i + 1). Finally, SampEn is calculated as the negative logarithm of the conditional probability that two similar sequences of i data points will be similar for the next i + 1 points:

(1) $$\mathrm{SampEn}\left(i\right)=-\ln\frac{N\left(i+1\right)}{N\left(i\right)}$$

Therefore, two sequences are considered to be similar if the differences between each of the paired data points of the two sequences (i.e., N(i) and N(i + 1 )) fall within the range of *r*. Traditionally, *m* is fixed to the value 2, and *r* as 15% of the *SD* of the original time series that is being analyzed. Therefore, for the present work, we applied these parameter settings.

#### 2.4.4. Interpreting MSE Time Scales

As mentioned above, theoretically, small/fine time scales in MSE reflect local neural interactions, while large/coarse time scales reflect the activity of widely distributed neural networks (Grundy et al. 2017; Vakorin et al. 2017). Linear stochastic effects are assumed to be related to observational noise at lower time scales. Coarse-graining applied during MSE analysis is essentially a down-sampling procedure that alleviates linear effects in large time scales. In this way, small-timescale MSE extracts information from the whole frequency spectrum and also captures linear stochastic effects in the signal, while large-timescale MSE relates to slow oscillations and reflects non-linear signal properties (Courtiol et al. 2016; Miskovic et al. 2019). The interpretation can be further simplified across the time domains. For example, in the present study, we computed MSE in the two experiments (i.e., verbal DT and the Simon task) for the time scales of 1–20 and 1–10, respectively. Based on the sampling rate (250 Hz) used in the present work, the real-time sampling interval at scale 1 is 4 ms. Therefore, the MSE at scale 1 reflects the dynamic activities of the neural system at a resolution of 4 ms, which captures fast and slow dynamics. In a similar vein, scale 5 reflects the dynamical activities of the brain more slowly than a resolution of 20 ms, and scale 10 indicates activity at 40 ms resolution. At the largest scale of 20, the activity is at 80 ms resolution and reflects slow brain dynamics. Thus, at smaller time scales, MSE mainly reflects fast and, hence, local neural activities, whereas, at larger scales, MSE mainly captures slow dynamics across broader spatial domains. However, a new study by Kosciessa et al. (2020) argues against such a traditional interpretation of MSE time scales, based upon direct scale-to-frequency mapping. The study demonstrated that entropy at fine time scales is highly sensitive to broadband spectral power, which is dominated by the low-frequency contribution. This new line of work challenges the implementation of the similarity-bounds *r* (by which the signal *SD* is multiplied). Traditionally, *r* is not equally liberal across all time scales because it is used as r*SD of the original time series, i.e., r*SD will be used for all time scales. As Kosciessa et al. (2020) demonstrated, this approach leads to biased entropy estimation. The signal during MSE analysis is successively coarse-grained at different time scales, except in the case of scale 1, which is similar to low-pass filtering. The value for *r* is only calculated relative to the *SD* of the original, unfiltered signal (at scale 1). However, successive coarse-graining reduces the *SD* of the signal and, as a result, signal variance is normalized; this introduces biased entropy estimation if it were calculated with a constant *r*. Therefore, the authors suggest computing the similarity bounds for each scale factor. Following these suggestions, we additionally performed global similarity bounds or scale-wise *r* MSE analysis. The results are provided in Supplementary Material S1. They reveal that the traditional MSE and scale-wise *r* MSE did not considerably differ in this particular application. Therefore, the multivariate analyses reported in the main part of the paper were based upon traditional MSE analyses, using invariant-*r* (or global similarity bound), because, as such, the results can be directly compared with the results of the previous study (Kaur et al. 2020).

#### 2.4.5. MSE Computation Using the EEG Signals Acquired during the Verbal DT and the Numerical Simon Task

The lengths of trials in the verbal DT task (typical and original associations) varied from trial to trial and from person to person (for more details, see Kaur et al. 2020). The variation across the trials was due to the non-restricted response time. Figure S2.1 in the Supplementary Material S2 provides a visualization of the average reaction time across all trials, during typical and original associations, and across participants. The figure shows that individuals were taking a variable amount of time to produce the associations. We aimed to capture the complete idea-generation process; therefore, the signal across all trials of varied length was concatenated separately for each condition and each participant. The MSE scores calculated during the production of typical associations are referred to as MSE in fluent neural states; MSE calculated during the production of original associations are referred to as MSE in creative thinking neural states. The same procedure was applied for the Simon task. The length of the trials varied but, due to frequent compatible and fewer incompatible trials (see the average reaction time across compatible and incompatible trials and participants in Figure S2.2 in the Supplementary Material S2), it was critical to standardize the length across the two conditions. Therefore, we concatenated the trials of the EEG time series for each condition and then selected 2500 data points (10 s) from each condition. The MSE scores were calculated for the concatenated incompatible trials, which were labeled as MSE in inhibition and, for the concatenated compatible trials, were labeled as MSE in non-inhibition. These neural indicators of inhibition were calculated for each participant, at each electrode, across multiple time scales (ranging on a scale of 1 to 10). The trial length in the Simon task was short. This is the reason for only calculating MSE in this task up to time-scale 10. As systematically illustrated by Li et al. (2020), the reliability of MSE decreases with the decreasing length of the time series. Thus, for these rather short time series, a higher-scale MSE would not satisfy these reliability expectations.

#### 2.4.6. Integrated MSE Scores Using the Area under the Curve (AUC) Measures

MSE analyses provide entropy values for each electrode and time scale. Thus, for statistical analyses, it is advisable to integrate these values, given that their interpretation is equivalent. Hence, we integrated MSE values across several scales into a single score by using the area under the curve (AUC). This integration procedure has been previously proposed in the literature because low and high scales are interpreted differently but interpretations do not differ for neighboring scales (see Kaur et al. 2019; Li et al. 2020; Takahashi et al. 2009). For a data-driven division of time scales in high and low results in the case of the verbal DT task, we visually inspected the line-plots of grand-mean MSE during the production of typical vs. original associations (see also Kaur et al. 2020). The line plots suggested that the MSE difference between the two experimental conditions increases across small scales (1–5), shows a rather stable condition difference at medium scales (6–15), but shows no difference at large scales (16–20). Therefore, we divided the timescale-specific MSE values into three categories: small-scale MSE, ranging from scales 1 to 5; medium-scale MSE, including scales 6–15, and large-scale MSE from scales 16 to 20. We integrated the person- and condition-specific MSE values by summing them up across those scales, resulting in three AUC scores: small-, medium- and large-AUC scores for every individual and condition. For the Simon task, we categorized the scales into small (1–5) and medium time scales (6–10) (see grand-mean MSE line-plots of the Simon task in Figure S3, Supplementary Material S3).

#### 2.4.7. Statistical Analyses

We applied path modeling by using the R Software for Statistical Computing (R Foundation for Statistical Computing 2013). For model estimation, we used the lavaan (latent variable analysis) package by Rosseel (2012). Given the small sample size of this study, structural equation modeling (SEM) results on the behavioral data are not reported in the main part of the manuscript. However, for completeness, SEMs are reported in the supplement. Because the path models were saturated, model fit is not assessed for these models. For SEMs in the supplement, the following test statistics and fit indices are reported: the chi-square fit statistic (ꭕ2), the comparative fit index (CFI, which should exceed 0.95 for a good fit), standardized root mean square residual (SRMR, to be lower than 0.08), and root mean square error of approximation (RMSEA, to be lower than 0.08). Missing data were handled by the full information maximum likelihood algorithm, as implemented in lavaan.

## 3. Results

### 3.1. Relationship between Creative Thinking, Intelligence and Inhibition

**Figure 1:**
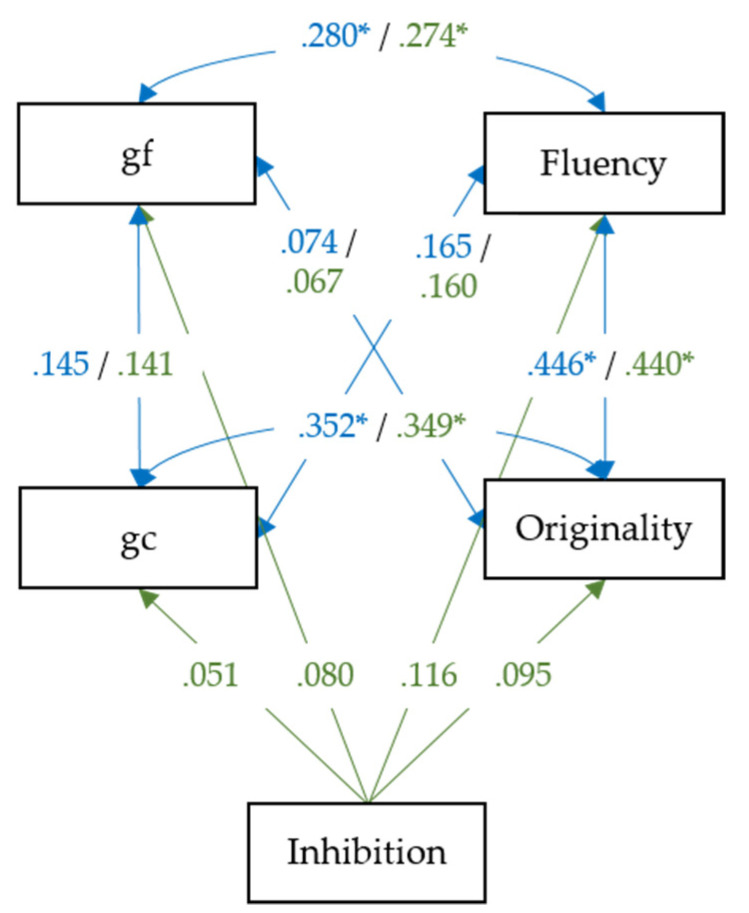
Path models, estimated to investigate the relationship between creative thinking, intelligence, and inhibition. The numbers are color-coded to indicate parameter estimates from the two different models. Blue-coded parameter estimates belong to Model 1 and green-coded ones to Model 2. Significant associations are indicated by the asterisk sign (*, *p* < 0.05).

We estimated two path models to examine the relationship between creative thinking, intelligence, and inhibition. In Model 1, the behavioral scores of gf, gc, fluency, and originality were used to estimate six correlations between the observed variables, as illustrated in Figure 1. In Model 2, the correlated gf, gc, fluency, and originality scores were then regressed onto the observed inhibition score. Figure 1 illustrates the model structure with parameter estimates, including correlation and path coefficients estimated via Model 1 (blue) and Model 2 (green).

The fluency variable is an average score of the four fluency tasks: SA, IN, FF, and RF. The originality variable is obtained by averaging the scores of the CO and NI tasks. gf is obtained by averaging the scores of gff and gfv, and gc is an average of the scores of gcnature, gchuman, and gcsocial. The inhibition score is the mean reaction time difference between the inhibition and non–inhibition trials of the Simon task.

Moderate and statistically significant relationships were observed between fluency and originality (*r* = 0.446, *p* < 0.001), and originality and gc (*r* = 0.352, *p* = 0.008). Weak and non-significant associations occurred between gf and originality (*r* = 0.074, *p* = 0.556), and gc and gf in the present small sample (*r* = 0.145, *p* = 0.256) and at the level of observed variables. Finally, gc and fluency showed a weak, statistically non-significant association (*r* = 0.165, *p* = 0.197). In Model 2, gf, gc, fluency, and originality were regressed onto inhibition. The inhibition variable did not predict gf (*ß* = 0.080, *p* = 0.522), gc (*ß* = 0.051, *p* = 0.684), originality (*ß* = 0.095, *p* = 0.447), and fluency (*ß* = 0.116, *p* = 0.355). Given the small sample size of this study and, potentially, the single-task approach for measuring inhibition, none of the associations with inhibition were substantial. Some associations between intelligence and creative thinking variables slightly changed when controlling for inhibition (see Figure 1). The association between fluency and originality, and gf and fluency, slightly decreased. In conclusion, at the behavioral level, we do not find support for the explanatory role of inhibition in the intelligence–creative thinking association. Potential reasons will be discussed. Next, we aim to explore the relationship between inhibition, intelligence, and creative thinking at the neural level.

Note that we also estimated two SEMs for the behavioral indicators of creative thinking and intelligence (see the model estimation and fit results in the Supplementary Material S4). SEM allowed us to estimate associations at the level of latent variables that are adjusted for measurement error. More importantly, SEM allowed quantifying individual differences (as factor scores) in order to investigate brain–behavior associations on that basis, instead of simple task-average scores. Even if underpowered, the SEM-derived factor scores provide descriptively better indicators for abilities previously reported in the literature at the latent level (Benedek et al. 2014; Frith et al. 2021b).

### 3.2. MSE as a Neural Marker of Inhibition

**Figure 2:**
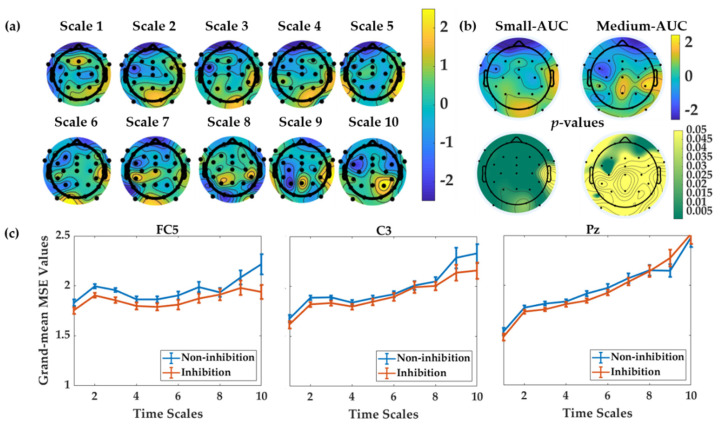
Illustration of the MSE difference between inhibition and non-inhibition conditions. Panel (**a**) shows the topographic plots of MSE difference scores between the two conditions across 10 time scales. The color bar indicates standardized MSE values on a *z*-scale. The yellow color indicates positive differences, and the dark blue, negative differences. The top row of panel (**b**) displays the topographic plots of the grand-mean MSE difference between the two conditions for the small- and medium-AUC values. The bottom row shows the corresponding *p*-values of the difference scores. Panel (**c**) illustrates the grand-mean MSE line-plots for the two conditions across 10 time scales at three representative electrodes, namely, FC5, C3, and Pz. These electrodes were selected, based on the strongest difference exhibited in the single-scale MSE and the AUC-difference topographic plots and their statistical significance after correcting for multiple testing (dark green in the *p*-value topographic plots). Error bars represent standard errors of the mean.

To explore inhibition as a correlate of intelligence and creative thinking at the neural level, we first need to test whether MSE is a sensitive measure that can differentiate inhibition and non-inhibition states. Thus, to address this question across multiple time scales of MSE, we inspected the topographical pattern of the MSE differences between the two conditions across the scalp. For this purpose, we computed the difference for single-scale MSE measures, as well as the AUC scores between the two experimental conditions, by subtracting non-inhibition MSE from the inhibition MSE. In Figure 2, panel 2a provides the topographic plots of the grand-mean MSE difference between the two conditions across 10 time scales.

Generally, a positive difference (yellow-coded in Figure 2, Panel 2a) occurred at small time scales (1–4) in most central and parietal electrodes. At medium time scales (6–10), a positive difference is visible on the CP5, P3, Pz, CP6, and P4 electrodes. The top row of panel 2b displays the topographic plots of the difference between the two conditions for integrated small- and medium-AUC values. The bottom row represents the corresponding *p*-values of the difference scores in form of topographic plots. As indicated by these four plots, the most substantial differences between the two conditions across the scalp occur in the small AUC, compared to the medium AUC, for which the *p*-values do not survive correction for multiple testing. The small-AUC difference plot shows statistically significant differences across the entire scalp (as depicted in the dark green color of the *p*-value plots). The medium-scale-AUC difference plot shows only a few statistically significant effects at frontal electrode sites, with a considerably low *p*-value (< 0.005, corrected for multiple testing). Panel 2c illustrates the line-plots of the grand-mean MSE in the two conditions, for electrodes FC5, C3, and Pz. These electrodes were selected as examples. The line plots illustrate that MSE in the inhibition condition is reduced, compared with the non-inhibition condition across small time scales at the frontal and central electrode sites. This is in line with the notion that the dynamic neural system becomes more focused locally, with challenging information input (conflict compared with non-conflict trials; see also Kaur et al. (2019) for a systematic demonstration across different brain states as well as the discussion below).

### 3.3. MSE in Inhibition as a Correlate of MSE in Creative Thinking

**Figure 3:**
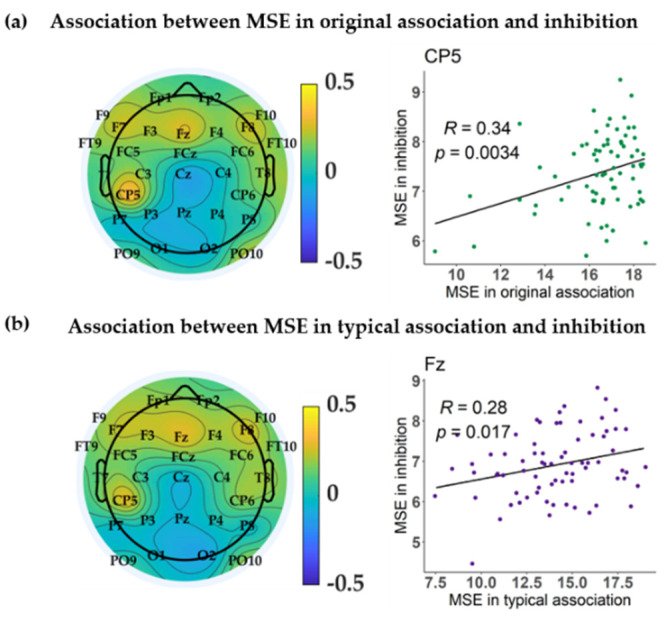
Associations between the grand-mean MSE in creative thinking and inhibition. The left topographical plot in panel (**a**) shows the Pearson correlations between medium-AUC values of MSE in the original association and small-AUC values of MSE in inhibition. Similarly, the left plot in panel (**b**) shows the Pearson correlations between medium-AUC in typical association, and small-AUC in inhibition. The yellow color on the topographical plots indicates positive correlations. The scatter plots on the right side of both panels illustrate the strongest and most substantial association at CP5 and Fz electrodes.

In Section 3.2, we demonstrated that MSE is a sensitive neural marker of inhibition at low time scales and in frontocentral electrode sites. Next, we investigated the relationship between inhibition and creative thinking at the level of neural signal complexity. For this purpose, we used the AUC scores of MSE, measured during tests of creative thinking vs. inhibition. A study by Kaur et al. (2020) showed the strongest difference between the typical and original association conditions at the medium time scales of MSE (see also Supplementary Material S1). Furthermore, as illustrated above, small time scales showed the most substantial differences between the inhibition and non-inhibition conditions across the entire scalp. Thus, we correlated AUC scores from the medium timescales of MSE in creative thinking and the small timescales of MSE in inhibition task conditions. Figure 3 illustrates these correlations in the form of topographic and scatter plots.

The left side of panel 3a shows scalp topographies of Pearson correlations between the respective AUC-MSE scores measured during original association, vs. the inhibition neural states. Similarly, the plot at the left side of panel 3b provides scalp topographies of Pearson correlations between MSE in typical association vs. inhibition neural states. As depicted in the topographies of both panels, moderate correlations are prominent in the frontal and left centroparietal sides of the scalp. Thus, positive and statistically substantial associations were observed at the electrodes F7, F8, Fz, CP5, and PO10. Additionally, the scatter plots on the right side of each panel depict the strongest associations between the neural measures of creative thinking and inhibition which were observed at CP5 and Fz electrodes, respectively. The scatter plots for all other electrodes at which the correlations were statistically significant are provided in the Supplementary Material S5. The above results are in line with our speculations that MSE in inhibition is positively associated with MSE in creative brain states at the frontal electrodes. The results further illustrate a slightly stronger and more robust association between inhibition and originality MSE (*r* = 0.34, *p* = 0.003) than between inhibition and fluency MSE (*r* = 0.28, *p* = 0.017).

Furthermore, we examined whether MSE in inhibition would explain the relationship between MSE in a creative vs. typical association. We thus computed partial correlations between the MSE in creative vs. typical association states, controlling for MSE in the inhibition condition. The observed correlation between the two conditions was high (*r* = 0.95, *p* < 0.001). After controlling for MSE in inhibition, their association did not drop substantially (*r* = 0.94, *p* < 0.001). For more details, see Supplementary Material S6. In conclusion, at the level of MSE associations, we do not find support for the explanatory role of inhibition in the association between fluency and originality. However, shared method-specific variance in the creative vs. typical association states but not in inhibition states might explain this negative finding.

### 3.4. On the Relationship between Grand-Mean MSE in Creative Thinking and Inhibitory Neural States, with Individual Differences in gf, gc, Fluency, and Originality

To explore further, we examined how MSE in creative thinking and inhibitory neural states are associated with individual differences in gf, gc, fluency, and originality. For this purpose, factor scores were estimated using the function lavPredict() in lavaan, which—in the case of quantitative data—relies on the Bartlett (1937) method. Factor scores are weighted by the factor-indicator association and are, thus, better ability estimates, compared with a simple average score. To explore the quality of factor scores, we computed the factor determinacy index (FDI) (Ferrando and Lorenzo-Seva 2018). The FDIs for each factor (gf, gc, fluency, and originality) are provided in the Supplementary Material S4. The results indicated that only the fluency factor reached an acceptable FDI score (a satisfactory value for research purposes is FDI > 0.80; see Ferrando and Lorenzo-Seva 2018). Nevertheless, we will use the factor scores instead of simple average scores because they are more appropriate for investigating brain-behavior associations, compared with non-weighted average scores. However, we refrain from strictly interpreting brain-behavior associations determined in this study as relationship estimates between MSE scores and latent abilities.

As shown previously, MSE in creative thinking and inhibition are moderately associated at certain scalp locations. To investigate relationships with performance measures and to settle on a region of interest (ROI) for testing these associations, we first explored the grand-mean MSE scalp distributions in creative thinking and inhibition (see the grand-mean MSE topographies of creative thinking and inhibition neural states across different time scales in the Supplementary Material S7). The topographies show that the average MSE across all participants in creative thinking and inhibition is the highest at the parietal electrode sites, at medium and large timescales. Therefore, based on these topographies, we selected a parietal ROI, including the P3, Pz, P4, P7, P8, O1 and O2 electrodes.

**Figure 4:**
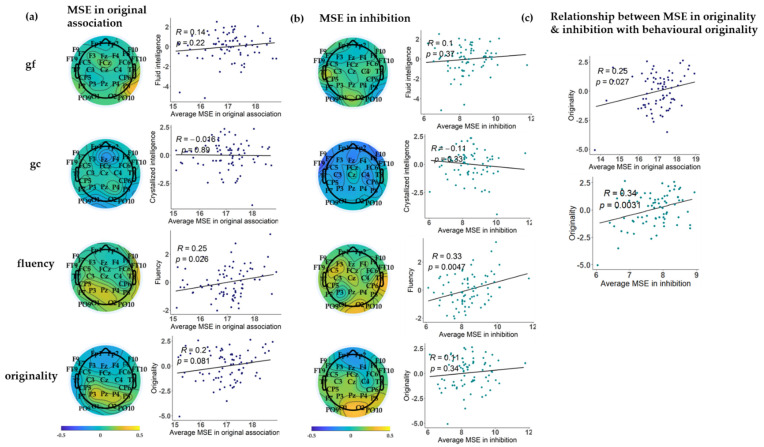
Correlations between grand-mean MSE in original associations and inhibition with gf, gc, fluency, and originality. The left topographical plots in panel (**a**) show Pearson correlations between medium AUC values of MSE in original association and factor scores of gf, gc, fluency, and originality. The scatter plots on the right side show the association between average MSE and AUC scores, based on the selected regions of interest and factor scores. The scatter plots illustrate the association between MSE in original association with fluency but not with gf, gc, and originality. Similarly, panel (**b**) shows Pearson correlations between small AUC values of MSE in inhibition and factor scores of gf, gc, fluency, and originality. The scatter plots on the right side illustrate associations only with fluency. The top scatter plot in panel (**c**) shows a small but statistically significant association between average MSE-AUC scores of originality at parietal ROIs (averaged MSE-AUC scores at P3, P4, and Pz electrodes, based on the topographies in panel (a)) with factor scores of originality. The bottom scatter plot shows a medium and statistically significant association between average MSE-AUC scores of inhibition at occipital ROIs (averaged MSE-AUC scores at O1 and O2 electrodes) with originality.

Next, for the purposes of exploration, we computed Pearson correlations between factor scores and medium AUC in the original association condition at every electrode. Similarly, Pearson correlations were calculated between factor scores and small AUC in inhibition across all electrodes on the entire scalp. Finally, for inferential tests, we computed correlations between AUC-MSE scores in the specified ROIs for both conditions and factor scores. In Figure 4, panels 4a and 4b provide topographical plots of the associations between MSE in both task conditions, with the computed factor scores. The correlations between typical association with the factor scores are provided in the Supplementary Material S8. The scalp topographies illustrate small to moderate positive associations at central-parietal sites for the fluency and originality scores but not for intelligence. The associations are, rather, located at parietal sites for MSE in the original association condition, whereas they are somewhat more central for MSE in the case of inhibition. The scatter plots on the right sides of both panels illustrate associations between the AUC-MSE scores in the ROIs and ability factor scores. The weak to moderate associations are statistically significant in the case of fluency and originality but not in gf and gc. However, as illustrated in panel (c) of Figure 4, the association between MSE in originality and the ability estimate of originality does not hold for the entire range of a-priori-specified ROIs but only for the P3, Pz and P4 electrodes. Similarly, MSE in inhibition and originality are moderately associated only at the occipital electrodes (O1 and O2).

## 4. Discussion

The present study aimed to explore the relationship between creative thinking, intelligence, and inhibition, specifically focusing on the BSC. Because no previous comparable studies exist, a hypotheses-generating approach seemed more appropriate. However, we had some a priori expectations with respect to the association patterns between these constructs. These were based on previous findings at the behavioral and neural levels, derived from adjacent neuroscientific literature on creative thinking measured by a verbal DT task, knowledge, and reasoning, as well as the temporal complexity of neural signals and their changes, depending on different task conditions (e.g., Kaur et al. 2019). A large number of association tests, based on the data at hand, only partially supported those expectations. Nevertheless, the described scalp patterns and magnitude of associations arguably provide valuable information for designing future confirmatory studies on neural complexity approaches to DT and intelligence, not only using MSE but also other signal complexity measures, such as, for example, non-oscillatory (1/f) signal components (see Ouyang et al. 2020). We will first summarize the present findings and discuss thereafter how they advance our knowledge about the neural dynamics underlying creative thinking, as well as the understanding of the link between facets of creativity and intelligence.

### 4.1. Summary of Findings

At the behavioral level, we replicated (for a review, see Silvia 2015) the positive creative thinking–intelligence relationship concerning gf and gc vs. fluency and originality. However, we could not replicate and extend the previous findings (Benedek et al. 2014), according to which inhibition partly explains the relationship between creative thinking and intelligence. Differently from previous studies, we had more comprehensive multivariate data at hand that included a larger number of tasks measuring different factors of DT and intelligence. However, we had a small sample size at hand, such that associations could not be tested at the latent level. Furthermore, the associations with cognitive control in the behavioral model failed to reach significance, potentially not only because of the sample size constraints but also because of the narrow assessment procedure for behavioral inhibition, which was measured with only one task in our study. Differently, Benedek et al. (2014) and Frith et al. (2021b) used multiple task paradigms to measure inhibition/switching ability.

At the neural level, we discovered that BSC can be considered a neural marker of inhibition. This was indicated by a quantitative difference between MSE in inhibition and non-inhibition neural states, especially at small timescales of MSE. Specifically, we found that the dynamical neural system responds with decreased complexity in local brain networks, to focus attention and deal with conflicting information when such input needs to be handled, given that an individual is confronted with a cognitive task. Further, we found that MSE in inhibition is correlated with MSE in original verbal association production but is also positively and moderately associated with MSE in typical verbal association production. Finally, we explored the associations between MSE in inhibition and fluent and creative thinking neural states, with individual differences in gf and gc, as well as fluency and originality. The findings revealed associations with MSE in creative and inhibition neural states, with fluency and originality but not with factors of intelligence. However, surprisingly, these occurred at parietal electrode sites and not at frontotemporal ones for which the neural complexity difference between creative and fluent association states was strongest, according to Kaur et al. (2020).

Taken together, our study extends the understanding of the creative thinking-inhibition relationship at the level of neural signal complexity, demonstrating the common involvement of neural inhibition states in fluency and originality. However, our neural complexity findings do not support the commonality of neural inhibition states and gf and gc. We will now elaborate on the above and point to potential future study directions.

### 4.2. Relationship between Creative Thinking, Intelligence, and Inhibition

Our study aimed at extending hitherto available findings on the explanatory role of inhibition, to understand the nature of the associations between creative thinking (fluency and originality) and intelligence (gf and gc). The four correlated variables of fluency, originality, gf and gc showed relationship patterns of different magnitude that were mostly in line with the previous literature but were smaller as they were estimated at the level of observed variables. Fluency and originality were most strongly associated among these four ability domains. The positive association between the two different factors of DT is in line with recent literature (for example, Weiss et al. 2020) in which fluency and originality are assessed in completely independent tasks with different instructions (“for fluency: create as many options as possible”; “for originality: create one, unusual option”). Other studies have also demonstrated a positive association between fluency and originality (Dumas and Dunbar 2014; Silvia 2008), but implemented different scoring methods. For example, in a study by Silvia (2008), the author reanalyzed Wallach and Kogan’s (1965) data in which the same items were scored for fluency and originality. Similarly, Dumas and Dunbar (2014) also used a single task to measure fluency and originality, in which fluency was scored as the average of the generated solutions and originality was parameterized by latent semantic analysis (LSA). When interpreting fluency vs. originality associations in the light of prior findings, it is important to note that the applications encompass different variants of scoring techniques, requiring complex human ratings such as average scoring (Benedek et al. 2014), top-two scoring (Silvia 2008), Wallach and Kogan’s (1965) uniqueness scoring (Silvia 2008), snapshot scoring (Jung et al. 2015), ratio quality scores (Forthmann et al. 2020), and residual scores (Runco and Albert 1985), to name a few. Furthermore, the literature has also criticized the measure of total creative performance due to its high correlation with the fluency of ideas and suggest that to evaluate the quantity and the quality of creative productions, researchers should study real creative outcome in terms of a linear function of quantity (e.g., Forthmann et al. 2021).

The present study—similarly to Weiss et al. (2020)—applied separate tasks for fluency and originality, with explicit instructions to produce fluent vs. original solutions. The item scores were averaged. According to this approach and by using multiple tasks for measuring fluency and originality, the correlation between the two abilities was high. The abovementioned studies, which applied different measurement and scoring techniques, yielded lower or even negative associations between fluency and originality. Our aim was to independently measure these ability domains and explicitly instruct individuals to be fluent or original. Given this approach, we learned that individuals who are fluent when they need to be fluent are also those who create more original solutions when uniqueness is expected. The high positive association between fluency and originality is theoretically justified because originality tasks require participants to produce the most creative solution; in order to do so, individuals first need to think of many potential solutions (fluency) and select the most original one with which to respond. Therefore, the more solutions a person could think of, the higher their chance to produce a solution that will be evaluated as creative by the raters. Furthermore, the literature suggests applying the consensual assessment technique (CAT) (Amabile 1982; Kaufman et al. 2013) for scoring, which has been described as the gold standard to be used for creativity ratings because it delivers highly reliable measures (Kaufman et al. 2013). The current study applied CAT. Thus, given the explicit “be creative” and “be fluent” instructions and applying established standard scoring techniques to independent tasks, we concluded that the distinct DT factors of fluency and originality are highly associated.

The association between fluency and gf was slightly lower at the observed level, compared to what has been typically observed in the literature (Beaty and Silvia 2013; Benedek et al. 2014). However, the weak association might be due to a larger measurement bias in a rather small sample size, compared with previous behavioral studies. At the latent level (see Supplementary Material S4), they showed moderate associations. Furthermore, gc was moderately associated with originality at the observed level (strongly at the latent level), indicating that the generation of creative ideas requires the adequate recombination of unrelated semantic concepts (Koestler 1964; Mednick 1962). gc is the ability that individuals accumulate through vocabulary and factual knowledge. Therefore, in verbal creativity tasks, especially when individuals are required to produce unique and context-appropriate answers, they need knowledge as a source for association generation. Individuals need to integrate various mental strategies that they would retrieve from relevant domain knowledge within a malleable problem space (Runco and Acar 2012). Therefore, gc assists by allowing individuals to retrieve knowledge from specific domains to generate original solutions. Hence, individuals will use their crystallized knowledge as a resource for the mental operations of creative thinking (Cho et al. 2010). However, Weiss et al. (2020) demonstrated that this correlation is canceled out if originality is nested under fluency. The large confidence intervals of the correlation indicate, however, that further investigations of this relationship are needed.

With respect to the explanatory role of inhibition in the creative thinking–intelligence relationship, we cannot derive strong conclusions based on the present behavioral data analyses. The results showed that after accounting for individual differences in inhibition, the association between gc and originality decreased to a small magnitude but, given the small sample size and a single task for inhibition measurement, none of the associations reached statistical significance. Thus, we could not replicate the finding that inhibition explains a significant part of the covariance between intelligence and creative thinking. An important reason might be that we used a single task to measure inhibition ability; however, this was not sensitive enough to capture the broader space of inhibition ability. Thus, for future studies, a more extensive measurement battery of inhibition should be involved to further explore the multivariate association between creative thinking, intelligence and cognitive control, going beyond hitherto-available studies (e.g., Benedek et al. 2014) by involving fluency as well as originality. However, psychometric issues in the domain of measuring cognitive control (see, e.g., Wilhelm et al. 2013) still need to be solved before these associations can be comprehensively tested in a latent variable modeling approach.

### 4.3. MSE as a Neural Marker of Inhibition

The MSE results in the Simon task can be interpreted in light of the dynamical system theory of complexity (Stam 2005), connected with the different levels of control (i.e., proactive and reactive control). The theory assumes that the state space of the signal is limited during visual input, compared to resting brain states with closed eyes (Kaur et al. 2019). In this framework, when individuals are continuously presented with homogeneous stimuli (i.e., non-inhibition/compatibility trials), the state space of the dynamical neural system is relaxed. While on the appearance of inhibition/incompatibility trials, individuals need to apply tonic control over automatic response activation, causing the system to be more focused. Therefore, a strong reactive control state would limit the state space, manifesting a lower entropy pattern.

Furthermore, MSE in inhibition was reduced across small time scales. The MSE time scales indicate different neural processing levels (McDonough and Nashiro 2014). Theoretically, small time scales represent local neural processing and accommodate information about the higher frequency components of neuronal activity, while coarser scales are related to global neural-level processing and slow neuronal oscillations (Courtiol et al. 2016; Grundy et al. 2017; McIntosh et al. 2014; Vakorin et al. 2017). Therefore, different time scales of MSE inform us about different neurophysiological mechanisms. Our results indicate that during inhibition, the system deals with conflicting information and responds with lower brain signal complexity in local neural networks. In contrast, MSE in non-inhibition is higher because the confrontation with compatible stimuli makes individuals alleviate their control state and disengage from proactive control, producing higher complexity patterns. To conclude, MSE is sensitive to the requirements of low and high control states and, therefore, can be considered an effective neural marker of inhibition-related brain states.

### 4.4. MSE in Inhibition as a Correlate of MSE in Creative Thinking

The moderate association between MSE in inhibition with MSE during creative thinking is in line with the notion that individuals need to inhibit their irrelevant responses to create novel compared to usual ideas. The lower MSE in inhibition states suggests that the system is more focused, and it generates lower complexity. However, the positive association between MSE in inhibition and in original verbal association production states might just represent stable individual differences in neural signal complexity across multiple states. Thus, the inhibition and creative thinking association needs further investigation at the level of difference scores (inhibition–non-inhibition and original–typical verbal associations) in the future, with more powerful studies in terms of sampling tasks and cases.

Here, we conclude that MSE in inhibition is positively associated with MSE in creative thinking neural states to some extent, and that this correlation might represent stable individual differences in neural complexity, independently of the cognitive state in which MSE is being measured. Our results revealed an association between MSE at coarser time scales, mostly at the frontal electrodes that were previously shown to be associated with information integration (Grundy et al. 2017). Therefore, the positive relationship between MSE in inhibition and creative thinking might express neural processing at the global level. Given the common involvement of the default mode network, the executive control network, and the prefrontal cortex in creative idea generation and inhibition (Chrysikou 2018), our results align with the literature, showing that creative idea generation and inhibitory control encompass widespread neuronal networks.

### 4.5. On the Relationship between MSE in Creative Thinking and Inhibitory Neural States, with Individual Differences in gf, gc, Fluency, and Originality

The present study is the first attempt to explore neural complexity estimates, measured in creative thinking and cognitive control neural states as correlates of intelligence and DT. Our exploration showed that individuals with higher BSC in creative thinking and inhibition neural states are better at producing fluent and original verbal associations. Thus, complexity in inhibition-related neural states is relevant for creativity but not for intelligence. We expected that MSE in inhibition is associated with creative thinking and intelligence as well, but only found robust associations with fluency and, at certain scalp locations, with originality.

As shown previously, MSE in original association and inhibition states are positively correlated. Furthermore, the spatial distribution of grand-mean MSE across the scalp was similar for both MSE in inhibitory and creative thinking neural states. We show that MSE in inhibition and creative thinking neural states show associations with behavioral fluency at scalp locations that are mostly parietal. Additionally, the magnitude of these correlations was also similar in both neural states. Therefore, these results provide a strong argument—now also from a neural signal complexity perspective—for the relevance of inhibition to creative thinking.

At the behavioral level, Benedek et al. (2014) showed that gf was predicted by updating but not by other executive functions (i.e., shifting or inhibition). Updating also showed a higher association with creative thinking, compared to inhibition. There is abundant literature showing that the updating facet of executive abilities is most strongly correlated with intelligence (Ackerman et al. 2005; Friedman et al. 2006), whereas inhibition has much lower associations. Updating and response inhibition have also been linked to creative thinking (Nusbaum and Silvia 2011), as well as fluency and flexibility (Benedek et al. 2012), but not originality. Therefore, the contribution of response inhibition to verbal creative thinking is less clear, even at the behavioral level. Furthermore, the MSE outcomes of creative thinking and inhibition herein described also suggest that the neural requirements involved in different types of tasks differentially affect MSE. Therefore, future studies—behavioral and neural complexity-related ones—will need to employ a broader measurement of executive function, such as updating, switching, and also controlled attention—ideally by measuring neural signal complexity in all these different brain states.

## 5. Limitations and Future Directions

The current study was mainly limited by its sample size. The association between creative thinking–intelligence–inhibition was lower in this sample, which might be due to a larger measurement bias in a rather small sample size. Furthermore, the assessment of behavioral inhibition was narrow, compared to the originality and fluency measurements. Therefore, future studies will need to employ a broader measurement spectrum of executive functions, including updating, switching, and controlled attention. Additionally, future research might investigate how the differences in the BSC measures of creative vs. fluent thinking and executive control vs. non-control states relate to creative thinking abilities. Response surface analysis (Humberg et al. 2019) might be a fruitful analytic approach to achieve this aim because it can overcome the shortcomings associated with difference score analysis. However, we first need to conduct robustness checks with respect to the regions of interest across the scalp and new data for replicating the associations discovered in this study.

Our conclusions are also limited to the human ratings of the DT performance. Such ratings, however, have been previously applied as the gold standard, despite several problems they imply (see, for example, Reiter-Palmon et al. 2019). Future studies could, however, benefit from computerized scorings, such as LSA (e.g., Prabhakaran et al. 2014). Such computerized scoring techniques still require validation in different languages, such as the German language. More knowledge about their comparability with human ratings is needed as well.

## 6. Conclusions

The present study demonstrated systematic individual differences in the EEG-derived MSE and behavioral performance outcomes, providing complementary insights into the neural foundations of the creative thinking–intelligence–cognitive control relationship, compared with what was previously known. Hitherto, the literature demonstrated the power spectrum in the alpha frequency band (or increment in EEG alpha power) as being a robust biomarker of creative ideation (Fink and Benedek 2014). Our study demonstrates that the non-oscillatory properties of the neural signal, such as BSC, measured by MSE, deserve additional attention toward a better understanding of the neural foundations of creative-thinking mental states. Furthermore, the study suggests that a multivariate approach to the assessed neural states is mandatory, involving, for example, not only creative verbal association states but also inhibitory and fluency-related mental states. This is because the BSC systematically differs across different tasks, and association patterns across these mental states need further convergent and discriminant validation, in order to establish non-oscillatory brain signal properties as biomarkers of intelligence and creative thinking. The present study has the potential to inspire complexity-based theories of creative thinking and intelligence and can guide the design of future multivariate studies.

## Data Availability

Data and scripts supporting reported results can be found under the link: https://osf.io/sg9e2/ (accessed on: 15 November 2021).

## Note

1 The task block analyzed in this study was part of a more comprehensive task procedure, which additionally consisted of another block with the reversed frequency of incompatible trials (i.e., mostly incompatible block, 80% incompatible and 20% compatible trials). The frequency manipulation of the trials was applied for a different research objective. Half of the participants started with the mostly incompatible followed by the mostly compatible block of trials. Importanty, for the present work, there were no between-group differences in reaction times to incompatible or compatible trials. Thus, we can conclude that the order of blocks did not influence the measure analyzed in this study.

## Supplementary Materials

The following are available online at https://www.mdpi.com/article/10.3390/jintelligence9040059/s1, Figure S1: Grand-mean MSE in typical (fluent) and original associations (original) in the verbal DT task; Figure S2: Average reaction times across all participants in fluent and original associations, and compatible and incompatible trails in the Simon task; Figure S3: Grand-mean MSE in inhibition and non-inhibition conditions; Figure S4: SEMs estimated to investigate the relationship between creativity, intelligence, and inhibition; Figure S5: Scatter plots of the associations between AUC scores of MSE in inhibition and creative neural states; Figure S6: Observed level correlation between MSE in originality and MSE in typical association; Figure S7: Grand-mean MSE in originality, inhibition, and fluent neural states for different time scales; Figure S8: Correlations between grand-mean MSE in typical association with behavioral gf, gc, fluency, and originality.
