# Supplementary material for: Exploring Neural Signal Complexity as a Potential Link between Creative Thinking, Intelligence, and Cognitive Control
Source: J Intell. 2021 Nov 30;9(4):59. doi: 10.3390/jintelligence9040059 (PMC8706335; doi:10.3390/jintelligence9040059)
Supplement: Supplementary file 1 [file jintelligence-09-00059-s001.zip › jintelligence-1209199-supplementary.pdf]

# Supplementary Material

## S1

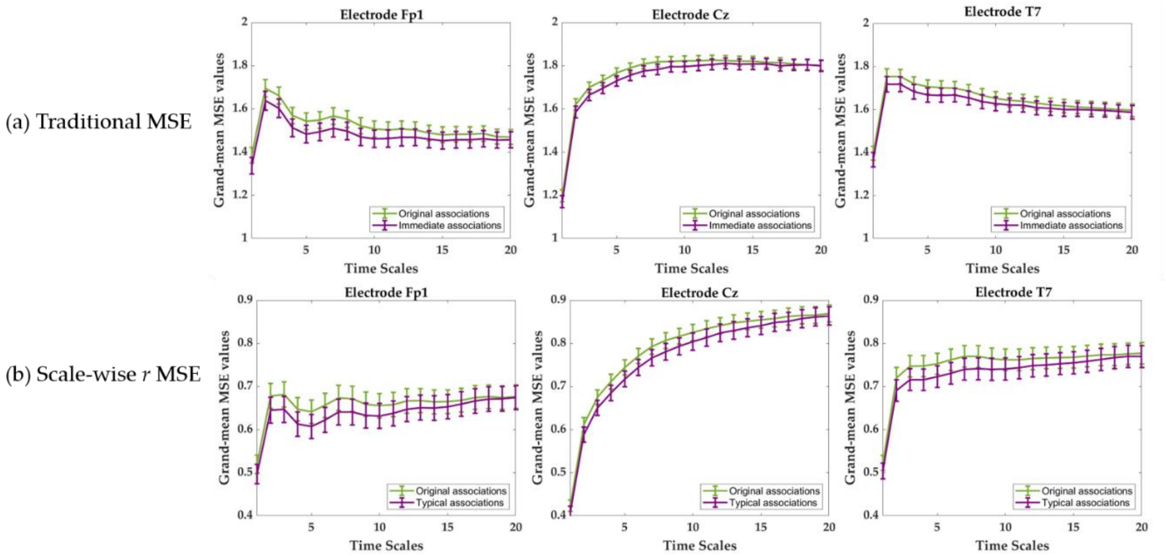


**Figure S1.** Illustration of the grand-mean MSE calculated in typical (fluent) and original associations (original) in the verbal DT task. Panel (a) shows the traditional MSE calculated with invariant similarity bounds (*r*). Panel (b) shows the scale-wise MSE computed with scale-wise *r*. The MSE is slight higher in the originality condition at smaller and medium time scales of the MSE in both panels.

## S2

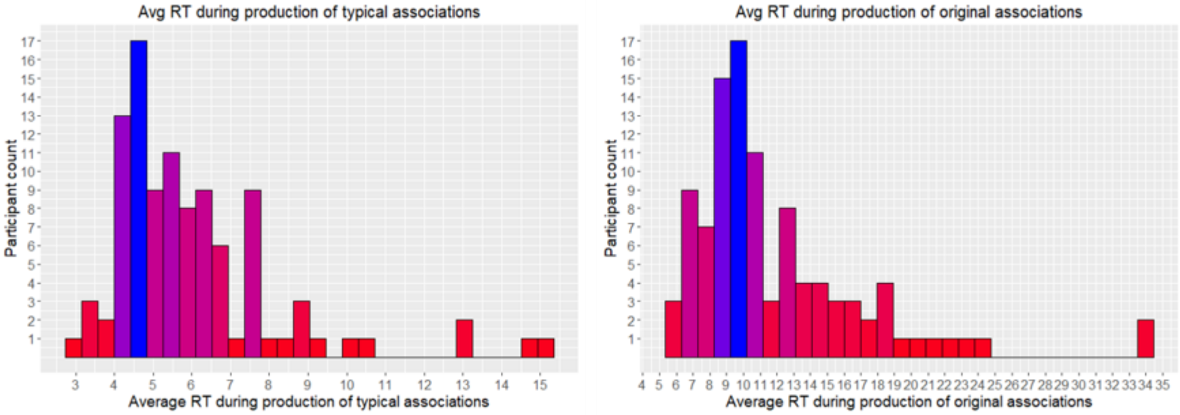


**Figure S2.1**: Distribution of average response times (Avg RT) in seconds across all participants required to produce typical vs. original associations. Different colors of the bars represent differential frequency in the data.

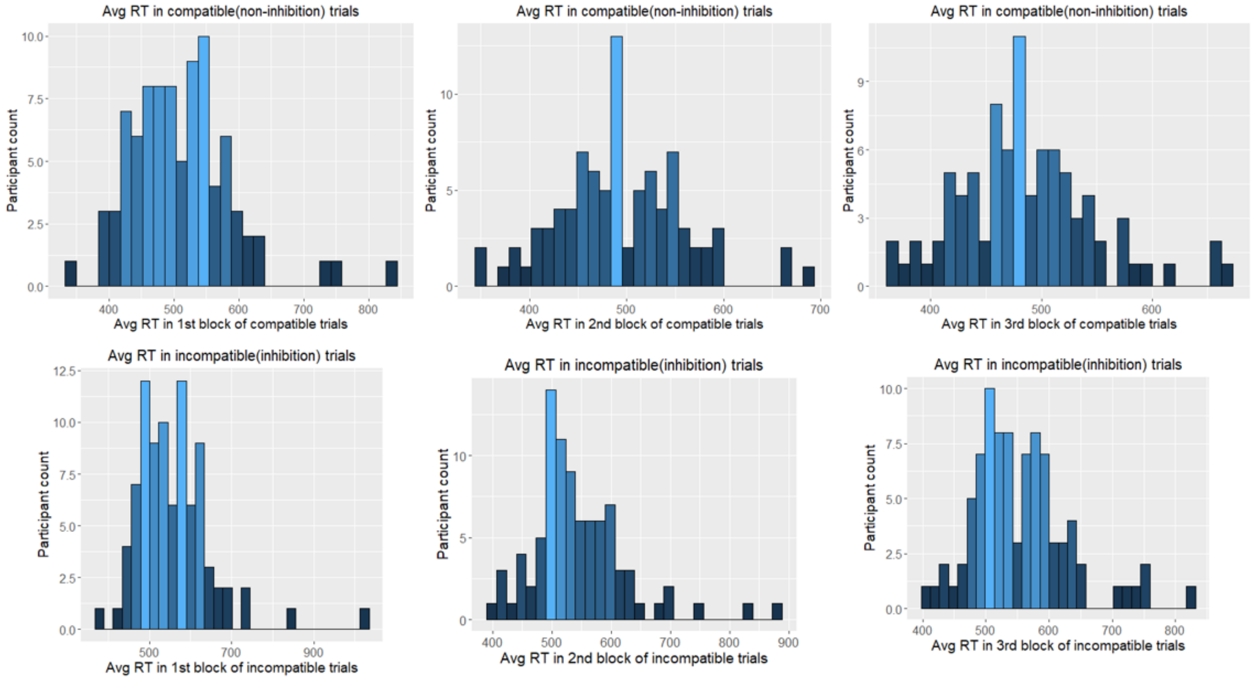


**Figure S2.2**: Distribution of average response times (Avg RT) in seconds across all participants required for compatible (first row) and incompatible (second row) trials in the Simon task.

# S3

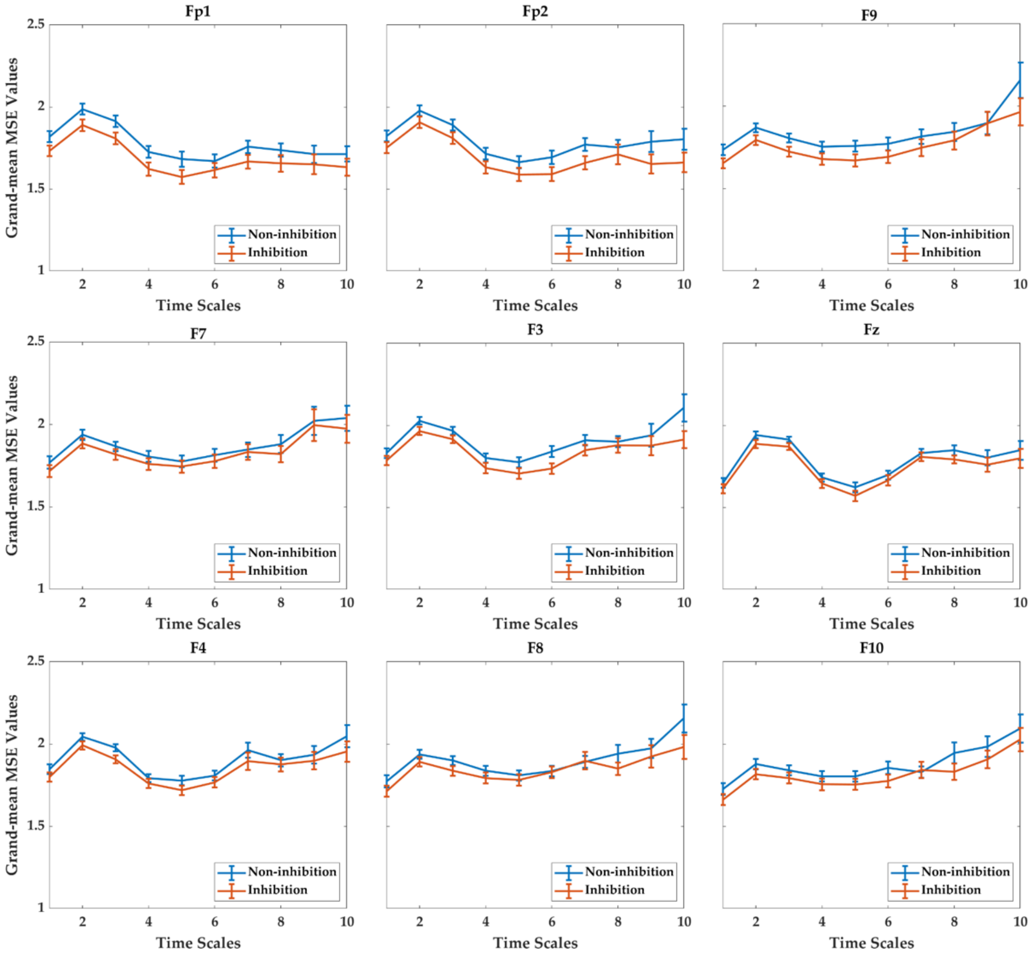

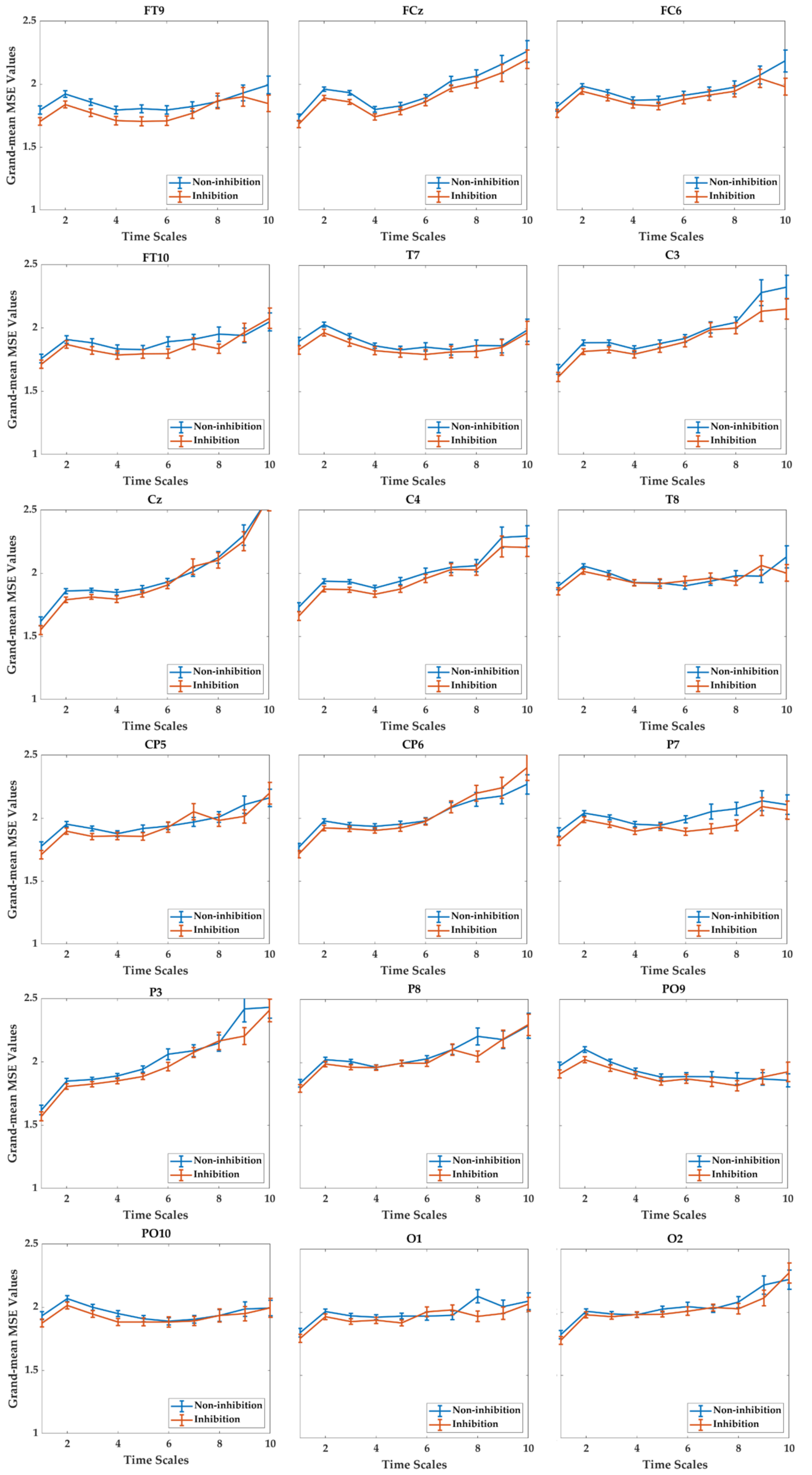


**Figure S3**: Grand-mean MSE in inhibition and non-inhibition conditions across 10 time scales and at remaining 27 electrodes. The MSE is slightly larger in the non-inhibition condition. Error bars represent 1 SE.

# S4 Individual Differences in Creative Thinking, Intelligence, and Inhibition

We estimated two SEMs to examine the relationship between creativity, intelligence, and inhibition. In Model 1, behavioral scores of gf, gc, fluency, and originality were used to estimate four correlated factors as illustrated in Figure S4. In Model 2, the correlated factors of gf, gc, fluency, and originality were then regressed onto a fifth inhibition factor. Figure 1 illustrates the model structure with factor loadings and latent correlations, as well as path coefficients estimated in Model 1 (blue) and 2 (green).

Model 1 had a very good fit: $\chi 2$ (40) = 40.54, $p = 0.44$, CFI = 0.996, SRMR =0.071, RMSEA = 0.013. The latent factors of gf and fluency showed a moderate positive relationship ($r = 0.347$, $p = 0.033$). Stronger relationships were observed between fluency and originality ($r = .849$, $p < .001$) and originality and gc ($r = 0.651$, $p = 0.030$). Weak and non-significant associations were observed between gf and originality ($r = 0.161$, $p = 0.510$) and gc and gf in the present quite small sample ($r = 0.258$, $p = 0.358$). Finally, gc and fluency showed a moderate, statistically significant association ($r = 0.373$, $p = 0.041$).

In Model 2 gf, gc, fluency, and originality were regressed onto inhibition. The fit was as follows: $\chi 2$ (57) = 55.96, $p = 0.514$, CFI = 1.00, SRMR = 0.073, RMSEA =0.00. The inhibition factor showed weak and non-significant associations with gf ($\beta = 0.189$, $p = 0.418$), gc ($\beta = 0.107$, $p = 0.732$), originality ($\beta = 0.269$, $p$ =0 .303), and negative on fluency ($\beta = -0.108$, $p = 0.586$). Given the small sample size of this study not even the inhibition-originality association reached statistical significance. However, when controlling for inhibition some association between intelligence and creativity factors slightly changed (see Figure S4). This is indeed no strong evidence, given that the associations between inhibition and the four factors were not substantial. In conclusion, at the behavioral level, we do not find support for the explanatory role of inhibition in the intelligence-creativity association.

The individual differences were quantified as factor scores which were obtained using the function lavPredict() in lavaan which for quantitative data relies on Bartlett (Bartlett 1937) method. Even if under-powered, the SEM derived factor scores provide descriptively better indicators for abilities. The quality of the factor scores was examined by the factor determinacy index (FDI; Ferrando & Lorenzo-Seva, 2018). The FDI scores for the factors are as follows: gf = 0.734; gc = 0.678; fluency = 0.918; originality = 0.649. It should however be noted that gf and originality factors have only two indicators each, and the gc factor has three heterogeneous indicators which both limit the FDIs.

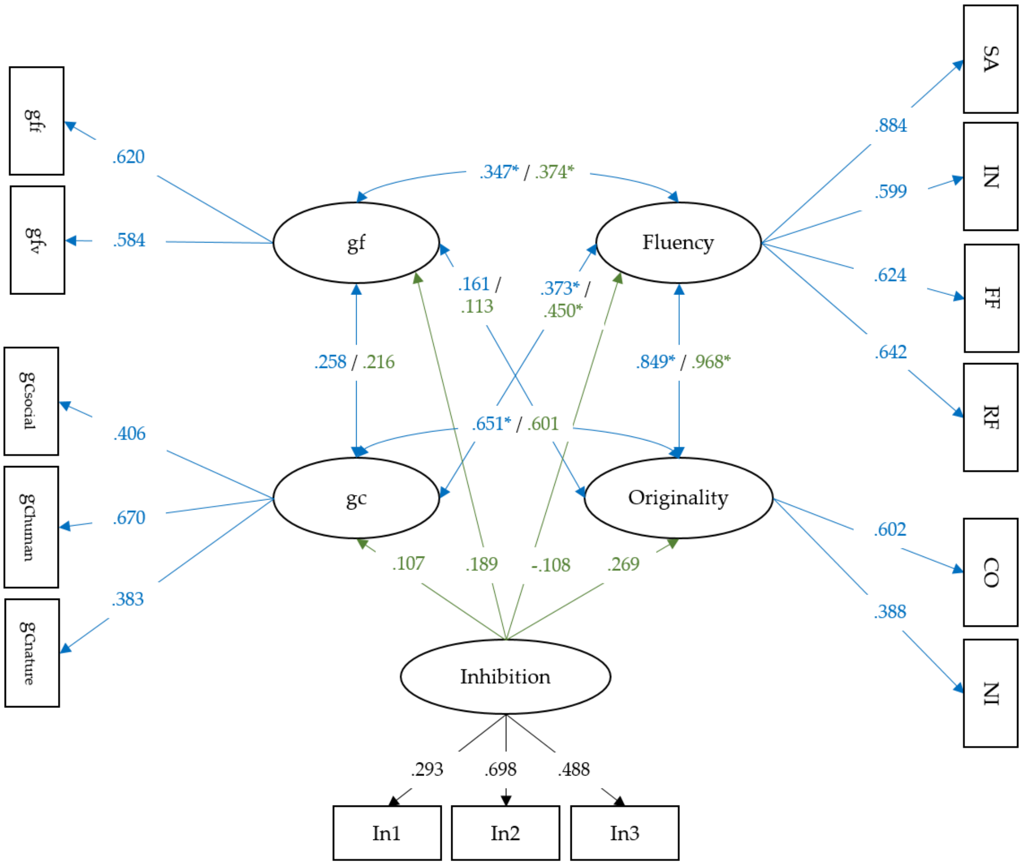


$X^2$ = 40.54 / 55.96, df = 40 / 57, CFI = .996 / 1.00, SRMR = .071 / .073, RMSEA = .013 / .000

**Figure S4.** Simplified schematic presentations of the SEMs estimated to investigate the relationship between creativity, intelligence, and inhibition. The numbers are color coded to indicate parameter estimates from the two different models: Blue coded parameter estimates and model fit indices belong to Model 1 and green coded ones to Model 2. Loadings are only provided for Model 1 as they did not considerably differ in Model 2. The latent factor fluency is measured by four indicators, similar attributes (SA), retrieval fluency (RF), figural fluency (FF), and inventing names (IN). The latent factor originality is measured by combining objects (CO), and nicknames (NI). Fluid intelligence (gf) has two indicators, figural fluid intelligence (gff), and verbal fluid intelligence (gfv). Crystallized intelligence (gc) tasks assessed knowledge in three domains natural sciences (gcnature, humanities (gchuman), and social studies (gcsocial). Inhibition was measured by three reaction time difference scores between the inhibition and non–inhibition conditions of the Simon task, labelled as In1, In2, and In3. Note that loadings on the gf and Originality factors were restricted to essential tau-equivalence for the respective factor. This is for the measurement model to be identified with two indicators only. Thus, non-standardized factor loadings of gff and gfv were restricted to equality, and CO and NI were fixed to be equal as well. As reflected in model fit, these restrictions were reasonable. Residuals are not displayed but were all estimated. Significant associations are indicated by the asterisk sign (*, $p < 0.05$).

## S5

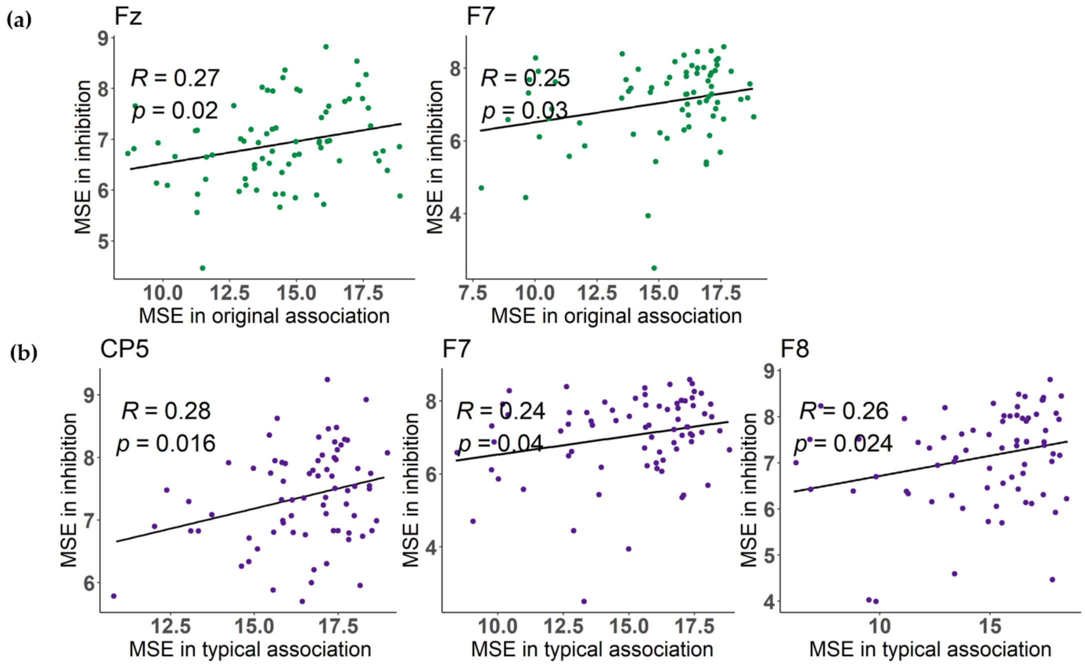


**Figure S5**: Scatter plots of the associations between AUC-MSE scores in inhibition and creative neural states. Panel (a) shows the correlation between small AUC-MSE scores in inhibition and medium AUC-MSE scores in originality related neural states, and panel (b) represents correlation between MSE in inhibition and fluency related neural states. The scatter plots for electrodes at which the correlations were statistically significant after correction for multiple testing involve Fz, F7, CP5, and F8.

## S6

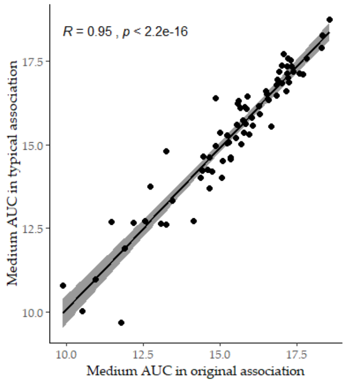


**Figure S6.** The correlation between MSE in originality (or creative neural states) and MSE in typical association (or fluent neural states). After controlling for the MSE in inhibition, the correlation was $r$ =0.94, t =25.44, df = 75, $p <$ 0.001 (partial correlation).

**S7**

## Grand-mean MSE in original association

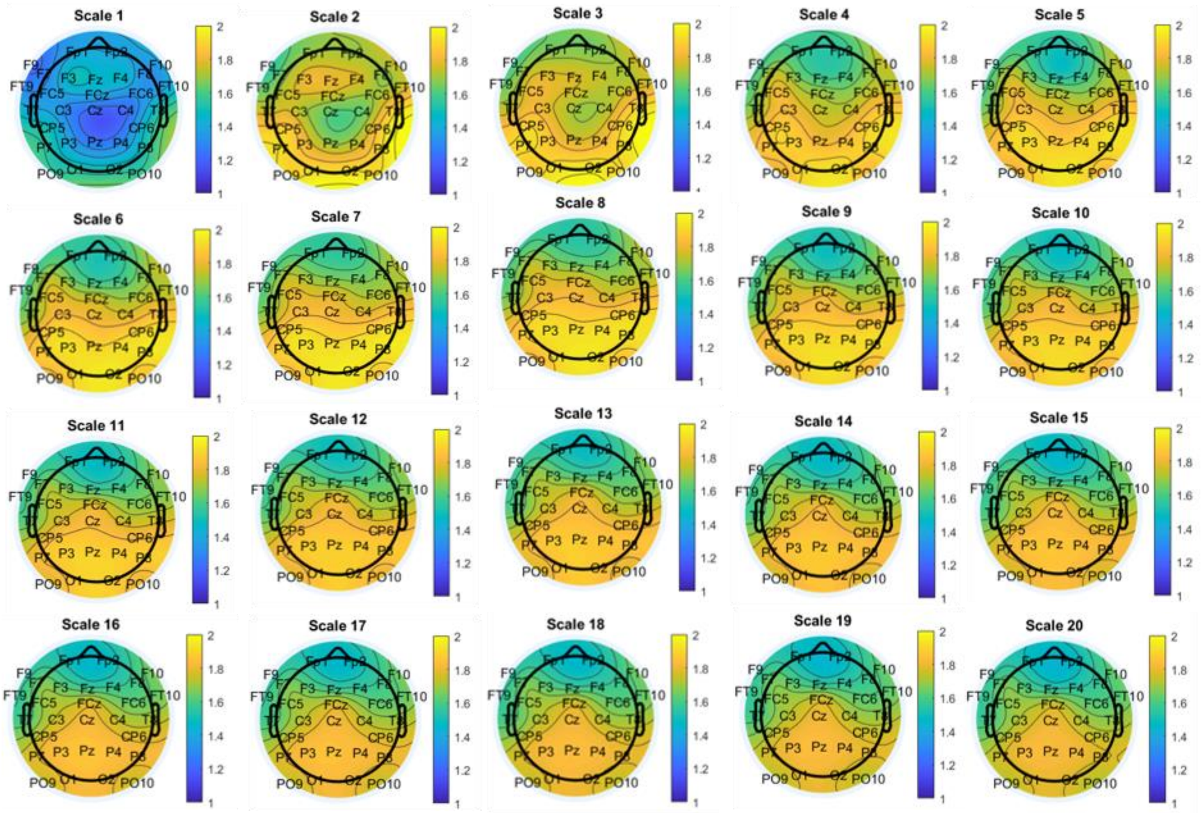


## Grand-mean MSE in inhibition

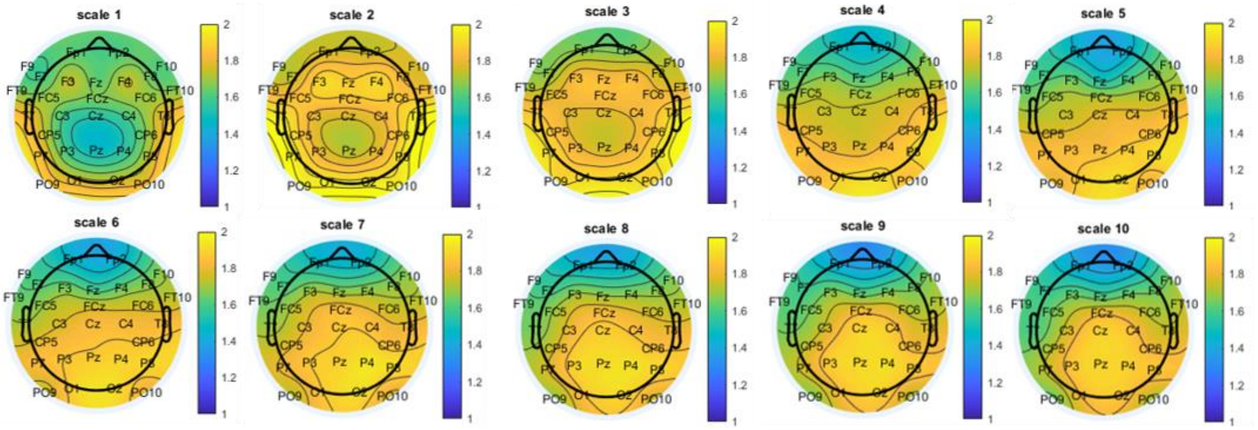

# Grand-mean MSE in typical association

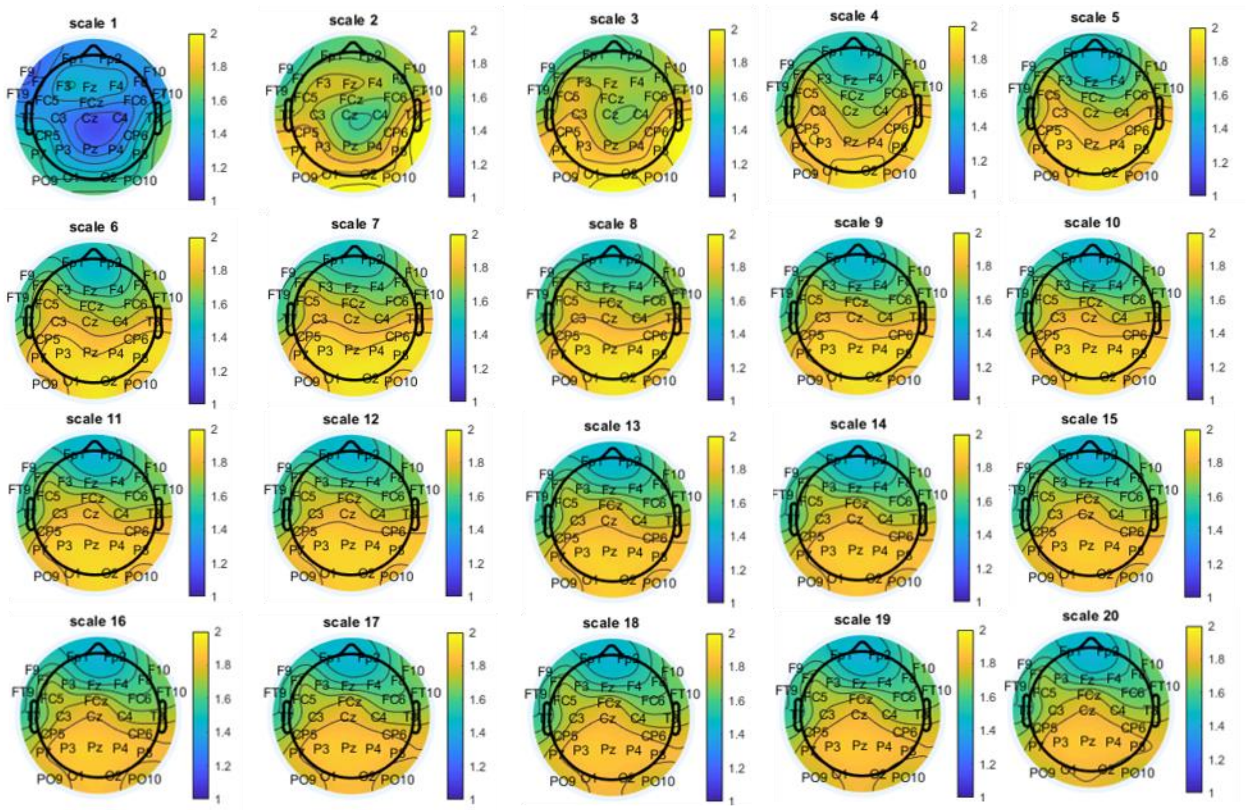


**Figure S7**: Grand-mean MSE in originality, inhibition, and fluent neural states for different time scales.

# S8

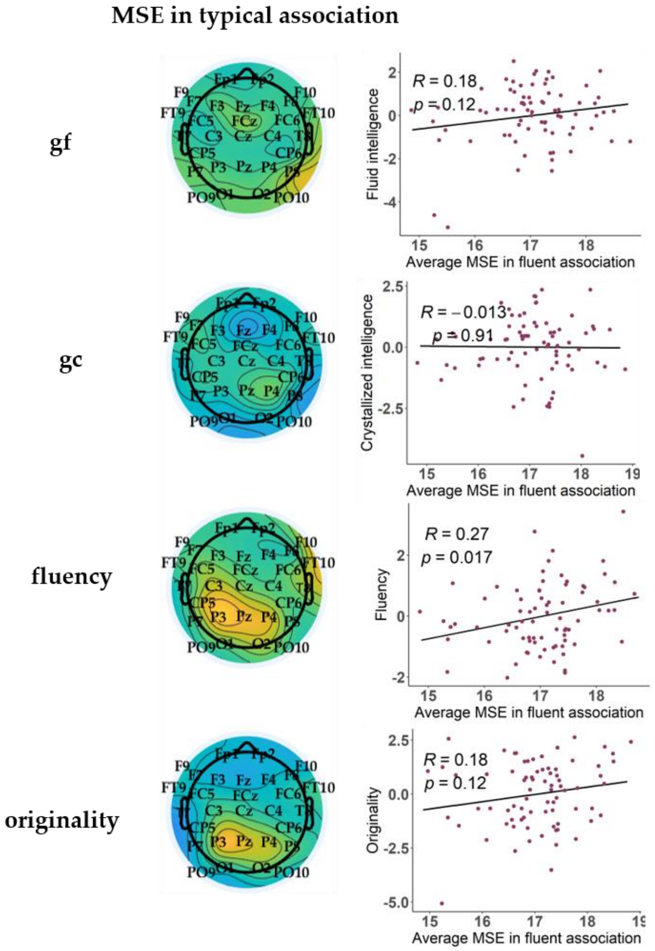


**Figure S8.** Correlations between grand-mean MSE in typical association with gf, gc, fluency, and originality. The left topographical plots show Pearson correlations across the scalp between medium AUC-MSE scores in typical association and factor scores of gf, gc, fluency, and originality. The scatter plots on the right side illustrate the association between average AUC-MSE scores based on the selected regions of interest and ability factor scores. The scatter plots illustrate a significant association between MSE in typical association condition only with fluency, given the pre-specified ROI. However, substantial associations occur with originality at the P3, Pz and P4.

## References


(Bartlett 1937) Bartlett, M. S. The Statistical Conception of Mental Factors. *British Journal of Psychology. General Section* 1937, *28* (1), 97–104. https://doi.org/10.1111/j.2044-8295.1937.tb00863.x.

(Ferrando & Lorenzo-Seva 2018) Ferrando, P. J.; Lorenzo-Seva, U. Assessing the Quality and Appropriateness of Factor Solutions and Factor Score Estimates in Exploratory Item Factor Analysis. *Educational and Psychological Measurement* 2018, *78* (5), 762– 780. https://doi.org/10.1177/0013164417719308.

(Kline 2015) Kline, R. B. 2015. *Principles and Practice of Structural Equation Modeling*, 4th ed: New York: Guilford Publications.

(Rosseel 2012) Rosseel, Y. 2012. Lavaan: An R Package for Structural Equation Modeling. *Journal of Statistical Software* 48: 1–36. https://doi.org/10.18637/jss.v048.i02.
